# Mitochondria-encoded peptide MOTS-c participates in plasma membrane repair by facilitating the translocation of TRIM72 to membrane

**DOI:** 10.7150/thno.100321

**Published:** 2024-08-19

**Authors:** Hong Jia, Lyu-Chen Zhou, Yong-Feng Chen, Wei Zhang, Wei Qi, Peng Wang, Xiao Huang, Jian-Wei Guo, Wai-Fang Hou, Ran-Ran Zhang, Jing-Jun Zhou, Da-Wei Zhang

**Affiliations:** 1 Department of Orthopedics, Xijing Hospital, Fourth Military Medical University, Xi'an 710032, China.; 2 Department of Physiology, Southwest Medical University, Luzhou 646000, China.; 3 Department of Neurology, Tangdu Hospital, Fourth Military Medical University, Xi'an 710032, China.; 4 Western Theater Command Center for Disease Control and Prevention, Lanzhou 730020, China.

**Keywords:** Mitochondria, Membrane repair, MOTS-c, TRIM72, Ischemia/reperfusion injury

## Abstract

**Rationale:** An impairment of plasma membrane repair has been implicated in various diseases such as muscular dystrophy and ischemia/reperfusion injury. MOTS-c, a short peptide encoded by mitochondria, has been shown to pass through the plasma membrane into the bloodstream. This study determined whether this biological behavior was involved in membrane repair and its underlying mechanism.

**Methods and Results:** In human participants, the level of MOTS-c was positively correlated with the abundance of mitochondria, and the membrane repair molecule TRIM72. In contrast to high-intensity eccentric exercise, moderate-intensity exercise improved sarcolemma integrity and physical performance, accompanied by an increase of mitochondria beneath the damaged sarcolemma and secretion of MOTS-c. Furthermore, moderate-intensity exercise increased the interaction between MOTS-c and TRIM72, and MOTS-c facilitated the trafficking of TRIM72 to the sarcolemma. *In vitro* studies demonstrated that MOTS-c attenuated membrane damage induced by hypotonic solution, which could be blocked by siRNA-TRIM72, but not AMPK inhibitor. Co-immunoprecipitation study showed that MOTS-c interacted with TRIM72 C-terminus, but not N-terminus. The dynamic membrane repair assay revealed that MOTS-c boosted the trafficking of TRIM72 to the injured membrane. However, MOTS-c itself had negligible effects on membrane repair, which was recapitulated in TRIM72^-/-^ mice. Unexpectedly, MOTS-c still increased the fusion of vesicles with the membrane in TRIM72^-/-^ mice, and dot blot analysis revealed an interaction between MOTS-c and phosphatidylinositol (4,5) bisphosphate [PtdIns (4,5) P_2_]. Finally, MOTS-c blunted ischemia/reperfusion-induced membrane disruption, and preserved heart function.

**Conclusions:** MOTS-c/TRIM72-mediated membrane integrity improvement participates in mitochondria-triggered membrane repair. An interaction between MOTS-c and plasma lipid contributes to the fusion of vesicles with membrane. Our data provide a novel therapeutic strategy for rescuing organ function by facilitating membrane repair with MOTS-c.

## Introduction

The plasma membrane undergoes damage during muscle contraction, particularly in eccentric exercise 1-3. It has also been observed in situations of mechanical stretching of pulmonary capillaries during osmotic stress and ventilation 4, as well as fluid shear stress of the blood vessel wall 5. Consequently, multiple hierarchies of repair machinery have been identified to promote cell function and survival 6, 7. A compromised or defective in membrane healing has been implicated in muscular dystrophy, myopathy, diabetes, and other diseases 8-11. Our recent studies have shown that the impairment of plasma membrane repair contributes to myocardial ischemia/reperfusion injury and severe burn-induced cardiomyopathy 12, 13. Therefore, uncovering the underlying mechanism will aid in preventing the progression of membrane-related diseases.

Several pathways have been reported to participate in membrane repair, including the fusion of intracellular vesicles with the plasma membrane, inter-vesicle fusion, caveolar endocytosis, and actomyosin contractions 6, 7. Regarding the molecular mechanism, TRIM72, also known as mitsugumin 53 (MG53), is a highly intriguing protein 14, 15. TRIM72 belongs to the tripartite motif family protein that comprises a really interesting new gene (RING) domain, B-box domain, and a coiled-coil domain at its N-terminus and a PRY/SPRY-contained domain at its C-terminus 15. The two protomers of TRIM72 interact with each other and pack in an antiparallel manner, which exhibit an elongated shape. The coiled-coil domain forms its two arms, and the B-box locates at the end of each arm. Under oxidizing stress, TRIM72 oligomerizes through C242 disulfide bond formation, and forms a high-order assembly when binding to the negatively charged phospholipid membranes, both of which help to nucleate vesicles and bring vesicles to the injured plasma membrane 16-18. TRIM72 exists in skeletal and cardiac muscle as well as nonmuscle tissues such as the lungs and kidney 19-21. Genetic ablation of TRIM72 (TRIM72^-/-^) leads to defects in membrane repair function in striated muscle and progressive skeletal myopathy 21. Whereas, exogenous recombinant human TRIM72 protein has been shown to enhance tissue regenerative capacity and protect against ischemia/reperfusion-induced muscle injury 14, 22, 23. In addition, dysferlin, caveolin3, polymerase I, nonmuscle myosin type IIA, and phosphatidylserine are also involved in the membrane repair process, and their functions are dependent on their interactions with TRIM72 24, 25. These findings highlight that TRIM72 is a critical nexus that confers an ability of a cell to reseal damaged membranes.

Mitochondria serve as critical signaling hubs within cells, which control cellular metabolism, stress response, and cell fate. However, the molecular mechanisms underlying these functions are not fully understood. MOTS-c is a 16-amino acid peptide encoded by mitochondrial DNA (mtDNA) and derived from the mitochondrial open reading frame of the 12S ribosomal RNA type-c 26. The first 11 amino acid residues of MOTS-c are highly conserved across 14 mammalian species 26. MOTS-c is expressed in muscles and other organs, while exercise is an important stimulator for MOTS-c generation 27. Accumulating evidence reveals that MOTS-c improves insulin sensitivity by modulating glucose and fatty acid metabolism 28, 29, transmits signals from the mitochondria to the nucleus 30, alters T-cell activation 31, ameliorates skeletal muscle atrophy 32, and increases physical activity 33. The downstream pathway of MOTS-c involves (1) increasing cellular levels of 5-aminoimidazole-4-carboxamide ribonucleotide (AICAR), subsequently activating AMPK 28; (2) binding to the promoter regions of nuclear factor erythroid 2-related factor 2-target genes possessing antioxidant capacity 30; (3) targeting the mTOR complex 31; and (4) inhibiting the transcription factor FOXO1 32. An intriguing observation is that MOTS-c passes through the plasma membrane into the plasma 29, 33, yet the pathophysiological relevance behind this process remains to be determined. We speculate that MOTS-c, as a messenger from mitochondria to the plasma membrane, facilitates the trafficking of TRIM72 to heal the damaged membrane.

In this study, we initially determined the correlation between MOTS-c and TRIM72 in humans to evaluate the association of MOTS-c with membrane repair. Subsequently, we investigated whether MOTS-c acts as a mediator between mitochondria and plasma membrane repair by using a high-intensity eccentric treadmill running model, characterized by plasma membrane disruption. Furthermore, we explored the role of MOTS-c in promoting membrane repair and its impact on TRIM72. Finally, we assessed the protective effects of MOTS-c against myocardial ischemia/reperfusion injury, a prevalent clinical condition. Herein, we identified a new role of MOTS-c in membrane repair, which involves MOTS-c/TRIM72 trafficking and the binding of MOTS-c to membrane.

## Materials and Methods

### Chemicals and reagents

Human-derived MOTS-c consisting of MRWQEMGYIFYPRKLR amino acid residues and the mutant of MOTS-c (MRWQEAGAAAAPRKLR) were synthesized by HbChinaPeptides (QYAOBIO, Hubei, China). Antibodies against MOTS-c (Cat# PAX132Hu01) and ELISA kits for MOTS-c (Cat# CEX132Hu) were provided by Cloud-Clone (Katy, USA). AMPKα1-siRNA (Cat# sc-29673), and Mouse and rabbit antibodies against TRIM72 were purchased from Santa Cruz Biotechnology (Cat# sc-514706, Shanghai, China) and Proteintech Group (Cat# 22151, Wuhan, China), respectively. Phosphatidylinositol (4,5) bisphosphate [PtdIns (4,5) P_2_] antibody (Cat# Z-A045) and PIP strips (Cat# P-6001) were purchased from Echelon (Utah, USA). The fluorescence probe Flipper-TR (Cat# SC020) and FM4-64 (HY-103466) was purchased from Spirochrome (Geneva, Switzerland) and MCE (Shanghai, China) respectively. Antibodies against clathrin (Cat# A4943), and GAPDH (Cat# AC033) were provided by Abcam (Shanghai, China) and ABclonal (Wuhan, China), respectively. TRIzol reagent (Cat# 15596026), PrimScript RT Master Mix (Cat# RR036), and TB Green Premix Ex Taq II (Cat# RR820) were purchased from Invitrogen and Takara, respectively. The primers and siRNAs were synthesized by GenePharma (Shanghai, China). Texa red^TM^-conjugated wheat germ agglutinin (WGA, Cat# W21405), secondary antibodies conjugated with tetramethylrhodamine (Cat# T2769), and AlexFluo®488 (Cat# A32723), Mem-PER^TM^ Plus Kit (Cat# 89842), Pierce^TM^ BCA protein assay kit (Cat# 23225), and Lipofectamine^TM^ 3000 (Cat# L3000015) were purchased from ThermoFisher (Illinois, USA). ATP assay kits (Cat# S0026) and cell counting kit-8 (CCK-8, Cat# C0037) were purchased from Beyotime (Suzhou, China). All other chemicals were analytical reagents provided by Macklin (Shanghai, China).

### Human participants

Participants were enrolled at Xijing Hospital, and this study was approved by the Ethics Committee of Xijing Hospital (no. KY20232009-F-1). Men or women between the ages of 18 and 80 who underwent orthopedic surgical treatment were eligible. All volunteers were informed about the research protocol, and all of them signed written consent forms. Subjects with systemic infectious diseases, autoimmune diseases, congenital developmental abnormalities, or who were currently participating in other medical clinical trials, pregnant, or breastfeeding were excluded. In addition, a questionnaire adapted from previous studies 34, 35 was completed by all subjects. It provided information on person-, work-, health-, and physical activity-related factors. The participants were classified into 2 groups based on WHO guidelines: moderate to vigorous physical activity, defined by ≥150 min of moderate-intensity or 75-150 min of vigorous-intensity physical activity per week, and low physical activity or sedentary behaviors, defined by <150 min of moderate-intensity 36, 37. Blood samples and excised skeletal muscles were collected before and during the surgical operation, respectively.

### Animals

Male wild-type C57BL/6 mice (23-25g) and Sprague-Dawley rats (250-300g) were obtained from the Animal Center of Air Force Medical University (Xi'an, China) and were kept in cages in a room with controlled temperature (22-24 ºC) and a light/dark cycle of 12/12 hours. TRIM72^+/-^ mice with deletion of exons 3-5 were provided by Shanghai Model Organisms (Shanghai, China). TRIM72^-/-^ mice were obtained by crossing heterozygous mice. All mice and rats were fed standard chow and water *ad libitum* and were allowed to adapt to the laboratory room for at least 3 days before the experimental procedure. All animal procedures were abided by *the Institutional Animal Care and Use Committee of Air Force Medical University,* and *the Guide for the Care and Use of Laboratory Animals (8^th^ edition)* issued by the National Research Council of the United States.

### Exercise protocol and exercise capacity test

Three days before exercise, mice were acclimated daily for 30 min on a nonmoving motor treadmill followed by 15 min at a velocity of 5 m/min. After this training period, mice were randomly allocated to four groups. (1) For the high-intensity exercise training group, a consecutive 7-day exercise training protocol with slight modification was adopted 33, 38. The treadmill had a decreased slope of 15º. Running exercise started at a speed of 5 m/min for 5 min to warm up, then increased to 13 m/min for 5 min, 18 m/min over a 30 min period, and 23 m/min until exhaustion. During treadmill running, an investigator continuously observed the mice to ensure that the appropriate running speed was maintained. Any mouse that resisted electric shock or prodding and remained on the platform for 30 seconds was considered to be exhausted. (2) In the MOTS-c treatment group, mice received high-intensity exercise training, and MOTS-c was administered by intraperitoneal injection at a dose of 15 mg/kg b.w. in the morning for 7 days. The dosage of MOTS-c was based on previous studies 33. On the other hand, the mice in the high-intensity exercise training group were injected with an equivalent volume of saline as a negative control. (3) In the moderate-intensity exercise training group, the mice ran at a constant speed of 8 m/min for 1 h. The other conditions were the same as those in the high-intensity exercise group. (4) In the sedentary group, mice were placed on a stationary treadmill for 1 h each day following a similar environment.

The day after completing the training, exercise capacity was assessed in a 30-minute running test. The treadmill was set at a speed of 23 m/min with a declined slope of 15º and was equipped with electrical shock to prevent animals from drifting back on the treadmill. During the test, the latency of the first shock to stimulate the animal was recorded. Mice remaining on the treadmill and refusing to run for more than 5s were considered to have failed the test. Exercise capacity was evaluated by the distance that the mouse ran and the latency of the first shock. Animals were killed by deep anesthetization (sodium pentobarbital, i.p. 150 mg/kg b.w.) after the last exercise session.

### Model of ischemia/reperfusion (I/R) injury

Rats were anesthetized with 3% pentobarbital sodium. A tracheostomy and a left-side thoracotomy were performed. Then, a 5-0 silk was placed around the left anterior descending coronary near its origin from the left coronary artery. The end of this ligature was passed through a small vinyl tube, and myocardial ischemia was produced by pulling the thread for 30 min. After ischemia, the ligature was loosened, and the ischemic myocardium was continuously reperfused for 120 min. Lead II electrocardiogram was recorded throughout the experiment. Ischemia was confirmed by ST-segment elevation on the electrocardiogram and a change in the color of the myocardial tissue around the ischemic area 39, 40.

Rats were randomly assigned to two groups: (1) I/R − intraperitoneal injection of 1 ml saline 30 min before I/R; (2) MOTS-c − intraperitoneal injection of 1 ml saline containing MOTS-c at 5 mg/kg b. w. 30 min before I/R.

### Myocardial performance

A PE50 catheter was connected to the right carotid artery to measure blood pressure and heart rate (HR) via a pressure transducer that was connected to a polygraph (RM-6200C, Chengdu, China). Then, the catheter was inserted into the left ventricular cavity to record the left ventricular pressure (LVP). Left ventricular developed pressure (LVDP), and the maximal positive and negative values of the instantaneous first derivation of LVP (±*dp*/*dt*max) were derived by the computer algorithms, and used to reflect myocardial performance, as described previously 41, 42.

### Determination of myocardial infarction

At the end of the experiment, the left anterior descending coronary was reoccluded, and the heart was perfused with 4 ml of Evans blue through the aorta to determine the left ventricular area at risk (AAR), which is defined as the area that doesn't turn blue. The heart was quickly removed and frozen at -20 ºC for 1 h. Then, the heart was cut into 1 mm-thin slices, and incubated with 1% 2,3,5-triphenyltetrazolium chloride solution for 15 min. The AAR, TTC-positive area (red staining, ischemic area), and TTC-negative area (white, infarct area) were analyzed with ImageJ software, and myocardial infarction (infarct area/AAR) was calculated 39, 40.

### Enzyme-linked immunosorbent assay and biochemical analysis

At the end of the test, the gastrocnemius muscle was homogenized with lysis buffer containing 50 mM Tris (pH 7.4), 150 mM NaCl, 5 mM EDTA, 5 mM DTT, and 1% Triton X-100. After centrifugation at 12 000 rpm for 30 min at 4 ºC, the supernatant was collected. Blood was collected from the eyes of the mice, and plasma was obtained from the blood by centrifugation at 3000 × g for 15 min at 4 ºC. The content of MOTS-c in plasma was measured with an enzyme-linked immunosorbent assay kit.

The level of creatine kinase, lactate dehydrogenase (LDH), and cardiac troponin I (cTnI) in plasma was measured with an automatic biochemical analyzer (AU480, Beckman Coulter, Brea, CA, USA), and the content of lactate was determined by the GEM Premier 3500 blood gas analyzer (Werfen, Barcelona, Spain).

To study the level of intracellular ATP, thirty micrograms of the gastrocnemius muscle was lysed with trichloroacetic acid, as described previously 43. The samples were reacted with an assay buffer provided by a bioluminescence detection kit, and the light output was read with a luminometer (VICTOR^TM^ X, PerkinElmer). The ATP content was calculated by a standard curve, and the data are expressed as nmol ATP/mg wet tissue weight.

### Culture of neonatal rat ventricular myocytes and hypoxia-reoxygenation protocol

Neonatal rat ventricular myocytes were isolated from hearts of 2-3 days old Sprague-Dawley rats by collagenase digestion as described previously 44. Briefly, hearts were removed under sterile conditions and ventricular myocytes were digested by 0.1% type I collagenase in calcium-free phosphate buffered-saline. Cardiomyocytes were pre-plated for 90 min in DMEM supplemented with 10% FBS containing 100 U/ml penicillin and 100 mg/ml streptomycin to reduce non-myocyte contamination and then plated in 6-well plate at 1 ×10^6^ cells/well and incubated at 37 ºC and 5% CO_2_ in humidified atmosphere. To induce cell injury, cells were exposed to hypoxia with a glucose-free Tyrode solution (pH=6.8) for 3 h. The O_2_ concentration was below 0.1% (Ohmeda Oxygen Monitor, Type 5120). After reoxygenation for 2 h, the cell viability was assessed with cellular ATP content and LDH concentration in the medium 44.

### Hypotonic solution exposure and assessment of plasma membrane damage

Mouse myoblasts (C2C12 cells) were cultured with Dulbecco's Modified Eagle Medium containing 10% fetal bovine serum and were differentiated into myotubes with 2% donor equine serum as previously described 45. After 3 d of differentiation, cells were exposed to hypotonic solution for 30 min, which consisted of (in mM) 10 HEPES (pH 7.4), 90 NaCl, 2 KCl, 1 MgCl_2_, 1 CaCl_2_, 10 glucose, and 30 mannitol, or exposed to isotonic solution as a control, which consisted of the same composition but with 110 mM instead of 30 mM mannitol 46. The values of both solutions, read by a vapor pressure osmometer, were 300 and 210 mOsm kg^-1^, respectively.

SiRNA was transfected with Lipofectamine 3000 to deplete either TRIM72 or AMPKα. Cells were transfected with 40 nM siRNA 6 h before the experiment 13. The sequence of siRNA-TRIM72 was 5′-GGGAGCTGTCAAGCCTGAACTCTTA-3′. Cell viability was assessed by counting the number of cells with CCK-8 according to the manufacturer's protocol, and the absorbance at 450 nm of each well was read. Propidium iodide uptake (100 μg/ml) was determined to allow imaging of plasma membrane damage 47. The fluorescence was excited at 543 nm, and the emission was recorded at 560-660 nm. Eight microscopic fields were randomly selected from each section, and 300 nuclei were counted. The membrane integrity was expressed by the percentage of propidium iodide-positive nuclei to the total nuclei.

To assess plasma membrane tension, FliptR was added to isotonic or hypotonic solution to reach a final concentration of 1 μM. Confocal microscopy equipped with an acoustic-optical beam splitter using gated hybrid detectors, a TimeHarp 300 TCSPC Module, and a Picosecond Event Timer (PicoQuant) was used to extract lifetime information. FliptR was excited with a pulsed 488 nm laser operating at 80 MHz, and the emission signal was collected at 549-651 nm. SymPhoTime 64 software (PicoQuant) was employed to fit fluorescence decay data. The photo histograms from membrane regions were fitted with a double exponential, and 2 fluorescence emission decay times (τ1 and τ2) were extracted. The longest lifetime and the higher fit amplitude τ1 were used to indicate plasma membrane tension 5, 48. The cells were examined from 5 min to 10 min after exposure to hypotonic solution. MOTS-c and its mutant at 1 μM were added 5 min before, along with the hypotonic solution.

### Plasmid construction, cell transfection and membrane repair assay

The coding sequence of TRIM72 (NM_001079932.3), TRIM72 N-terminus (1-284 amino acids region), and TRIM72 C-terminus (285-477 amino acids region), and the coding sequence of MOTS-c (KP715230.1) were synthesized by Tsingke company (Beijing, China). The cDNA of the full-length TRIM72, TRIM72 N-terminus, and TRIM72 C-terminus were cloned into the expression vector pLV4Itr-flag-ZsGreen-CMV vector by XhoI and BamHI restriction enzyme, and the cDNA of MOTS-c was cloned into pLV4Itr-myc-mCherry-CMV vector by XhoI and SmaI restriction enzyme. The sequences alignment for the cDNAs were carried out using DNAMAN software.

The role of TRIM72 truncates in the protective effects of MOTS-c against hypotonic solution was studied. In this scenario, human embryonic kidney 293T (HEK293T) cells were transfected with full-length TRIM72 or its truncates using lipofectamine 3000^TM^ following the manufacturer's protocol. Then, cells were exposed with hypotonic solution with MOTS-c, and cell viability was evaluated. When performing Co-IP, the plasmid expressing MOTS-c and the plasmid expressing full length TRIM72 or it truncates were co-transfected at a ratio 1.25 μg:1.25 μg per well in a 6-well plate.

Membrane repair assays were performed using a two-photon confocal laser-scanning microscope with ×40 magnification (FVMPE-RS, Olympus, Japan) coupled to a 1.85-W Mai Tai® EV laser, as described previously 21, 49. Briefly, after C2C12 cells were transfected with full-length TRIM72 or its truncates, a circular area with 5 μm in diameter was selected along the edge of the sarcolemma. Membrane damage was induced by 2 s irradiation at 20% maximal output power without or with 1 μM MOTS-c. Time-lapse images were captured using Fluoview FV31S-SW viewer software, which started at 3 s before (t=0) and for 60 s after irradiation at 3-s intervals. The green fluorescence intensity at the damage site (a square area with 10 × 10 pixels) was analyzed using ImageJ software to reflect membrane repair. In addition, the experiments were carried out in the presence of 2.5 mM FM4-64 dye (red color), and the red fluorescence was captured at the end of the last frame of green color scanning to determine membrane damage.

### Western blot and Co-immunoprecipitation (Co-IP)

Total protein was extracted from gastrocnemius muscle with a buffer containing (in mM): Tris (pH 8.0) 50, NaCl 150, EDTA 5, DTT 1, 1% Triton X-100, 0.5% SDS, and a cocktail of protease inhibitor. As described previously, the plasma membrane protein was prepared following the instructions provided by a Mem-PER^TM^ Plus Kit 13. Protein concentrations were quantified with a BCA protein assay kit. Each aliquot of protein from each sample was separated using 10% SDS-polyacrylamide gels before transfer to Immobilon® Transfer Membranes. After blocking, the membranes were incubated with TRIM72 (1:1000), MOTS-c (1:1000), and GAPDH (1:1000) overnight at 4 ºC, followed by incubation with horseradish peroxidase-conjugated secondary antibodies at room temperature for 1 h. Finally, the membranes were reacted with a chemiluminescent substrate, and the target protein was detected with a Quantity One system (Bio-Rad Inc, Herefordshire, UK). GAPDH and Ponceau staining were used as loading controls for total proteins and membrane proteins, respectively. The density of each band was analyzed by ImageJ software, and the data are expressed as the fold change relative to the control 13.

As described previously 12, a Co-IP study was performed to detect the interactions between MOTS-c and TRIM72. Briefly, a mixture of the tissue lysates and prewashed protein A-Sepharose beads were incubated with antibodies against MOTS-c (1:500), flag (1:500), or with normal IgG for the control group at 4 ºC overnight. Then, the beads were washed to remove nonspecifically bound proteins. After resuspension in the protein loading buffer, SDS-polyacrylamide gel electrophoresis was performed. MOTS-c, TRIM72 or flag antibody was used for immunoblot analysis. The homogenates without bead treatment were used as the input controls.

Dot blotting of a strip containing 15 different lipids was performed according to the manufacturer's instructions. Briefly, after blocking the strip at room temperature for 1 h with phosphate buffer containing 1% Tween 20 and 3% BSA, ten microliters of the sample at a concentration of 1 μg/μl was added onto the strip and incubated at room temperature for 1 h 21. After washing, the strip was incubated with MOTS-c antibody (1:1000) with agitation at 4 ºC overnight. Then, the strip was visualized with a Quantity One system.

### Total mRNA and DNA isolation and RT‒qPCR

Total RNA was extracted from the gastrocnemius muscle with TRIzol reagent. One microgram of total RNA was reverse-transcribed into cDNA with a Reverse Transcriptase Kit 50. Quantitative PCR (qPCR) was performed by using SYBR Green Premix Ex-Taq II with a CFX96TM Real-Time System (Bio-Rad, Hercules, California, USA), as described previously 50. The qPCR conditions were as follows: initial denaturation at 95 ºC for 30 s followed by 40 cycles of 5 s at 95 ºC and 34 s at 60 ºC. A total of 20 ng DNA was used in qPCR for the determination of the threshold cycle number (Ct). The following primers were used:

MOTS-c-F: 5′-GACACCTTGCCTAGCCACAC-3′,

MOTS-c-R: 5′-TGGCTGGCACGAAATTTACCA-3′ 50;

TRIM72-F: 5′-AGGTAGTTACAGGATGGGGCT-3′,

TRIM72-R: 5′-CATGGTGAGCCTGGGAAGAG-3′;

18S RNA-F: 5′-GCAATTATTCCCCATGAACG-3′,

18S RNA-R: 5′-GGGACTTAATCAACGCAAGC-3′.

Data were normalized to 18S RNA and analyzed by the 2^-ΔΔCt^ method. The results were expressed relative to the control group, which was represented as 1.

DNA was extracted using a phenol‒chloroform-isoamyl alcohol solution (25:24:1) followed by ethanol precipitation, as described previously 51. DNA was dissolved in 100 μl of Tris-EDTA, and 5 μl of a 50-fold dilution was subjected to qPCR using a SYBR Green PCR kit in a total reaction volume of 25 μl containing 0.5 μM of each primer. The sequences of the primers used were as follows:

cytochrome b-F: 5′-GTTCGCAGTCATAGCCACAG-3′,

cytochrome b-R: 5′-GGCGGAATATTAGGCTTCGT-3′.

Cyclophilin A-F: 5′-ACACGCCATAATGGCACTGG-3′,

cyclophilin A-R: 5′-CAGTCTTGGCAGTGCAGAT-3′.

The copy-number measurement of the mitochondrial gene (cytochrome b) relative to the nuclear gene (cyclophilin A) was calculated and used to indicate the mitochondrial copy number 51. Data were normalized relative to the control group.

### Confocal microscopy examination and quantification

The frozen sections were incubated with Texa red^TM^-conjugated WGA at 4 ºC overnight, or antibodies against MOTS-c, and TRIM72 at a dilution of 1:150 at 4 ºC overnight, which was followed by incubation with tetramethyl rhodamine-conjugated or Alexa Fluor®488-conjugated secondary antibodies at 1:500. After washing, the nuclei were counterstained with DAPI at 20 μg/ml. The sections were examined with a laser-confocal microscope equipped with an FV-10-ASW system (Olympus FV1000, Tokyo, Japan), as described previously 12.

To detect the plasma membrane integrity, Evans blue (10 mg/ml; 0.1 ml per 10 g body weight) was administered by intraperitoneal injection, and the mice were killed 24 h later 13, 45. Frozen cross-sections were prepared, and visualized under a laser-scanning confocal microscope with excitation and emission wavelengths of 543 nm and 590 nm, respectively 13, 45. With the use of ×60 magnification, 10 microscopic fields were randomly selected from each section, and examined by a histologist who was blinded to the group assignment. After the photomicrographs were taken using identical settings, the Evans blue-positive cells (red fluorescence signal) were counted using ImageJ software. The percentage of the positive cells to the total cells was calculated from each field and averaged.

For all the experiments involving immunofluorescence and membrane integrity, a minimum of ten serial sections in each of 3 separate regions per muscle from the mid-belly of the gastrocnemius muscle were analyzed.

### Transmission electron microscopy

Briefly, the gastrocnemius muscle was quickly excised at the end of the last session of exercise training and cut into blocks smaller than 1 mm^3^. The blocks were fixed with glutaraldehyde and OsO4 and embedded in Spur resin. Thick sections (50 μm) were cut, stained with toluidine blue, and examined to identify longitudinally oriented portions of the muscle in each block. Thin sections (70 nm) were then cut with an ultramicrotome (LKB Nova, Stockholm, Sweden) and mounted on copper grids. After counterstaining with uranyl acetate and lead citrate, the sections were examined with an electron microscope at 80 kV equipped with a Hitachi TEM system 43, 45.

After the full length of the myofiber was photographed, the plasma membrane was evaluated by an examiner who was blinded to the treatment group. Membrane with nanopores, unclear structure, or discontinuity was identified as injury. The percentage of damaged membrane length relative to the full length was calculated and averaged. In addition, mitochondria beneath the damaged membrane were counted and expressed as a ratio to the length of the damaged membrane. In this series of experiments, 3 separate regions per muscle from the mid-belly of the muscle were sampled, and a minimum of 20 myofibers in five sections were examined in each sample. Experiments were repeated in 8 animals.

### Statistical analysis

All data are presented as the mean ± standard error of the mean (mean ± SEM). GraphPad Prism (version: 8.4.2) was used to perform statistical analysis. The normality for continuous variables was checked by use of the Shapiro-Wilk normality test, and homogeneity of variance was examined with the Levene statistic test. Normal datasets for two independent samples were analyzed by an unpaired *t-*test, and comparisons among multiple groups were carried out using a one-way analysis of variance followed by Tukey's test. Datasets that did not have normal histograms were analyzed by the Mann‒Whitney U test for 2 independent samples and by the Kruskal‒Wallis H test for multiple groups. The frequency distribution among the categorical variables was assessed by chi-square analysis. Spearman's correlation test was used to determine the relationship between MOTS-c and TRIM72. A two-tailed *P* value of less than 0.05 was considered statistically significant.

## Results

### MOTS-c is related to membrane repair in human subjects

**Figure 1:**
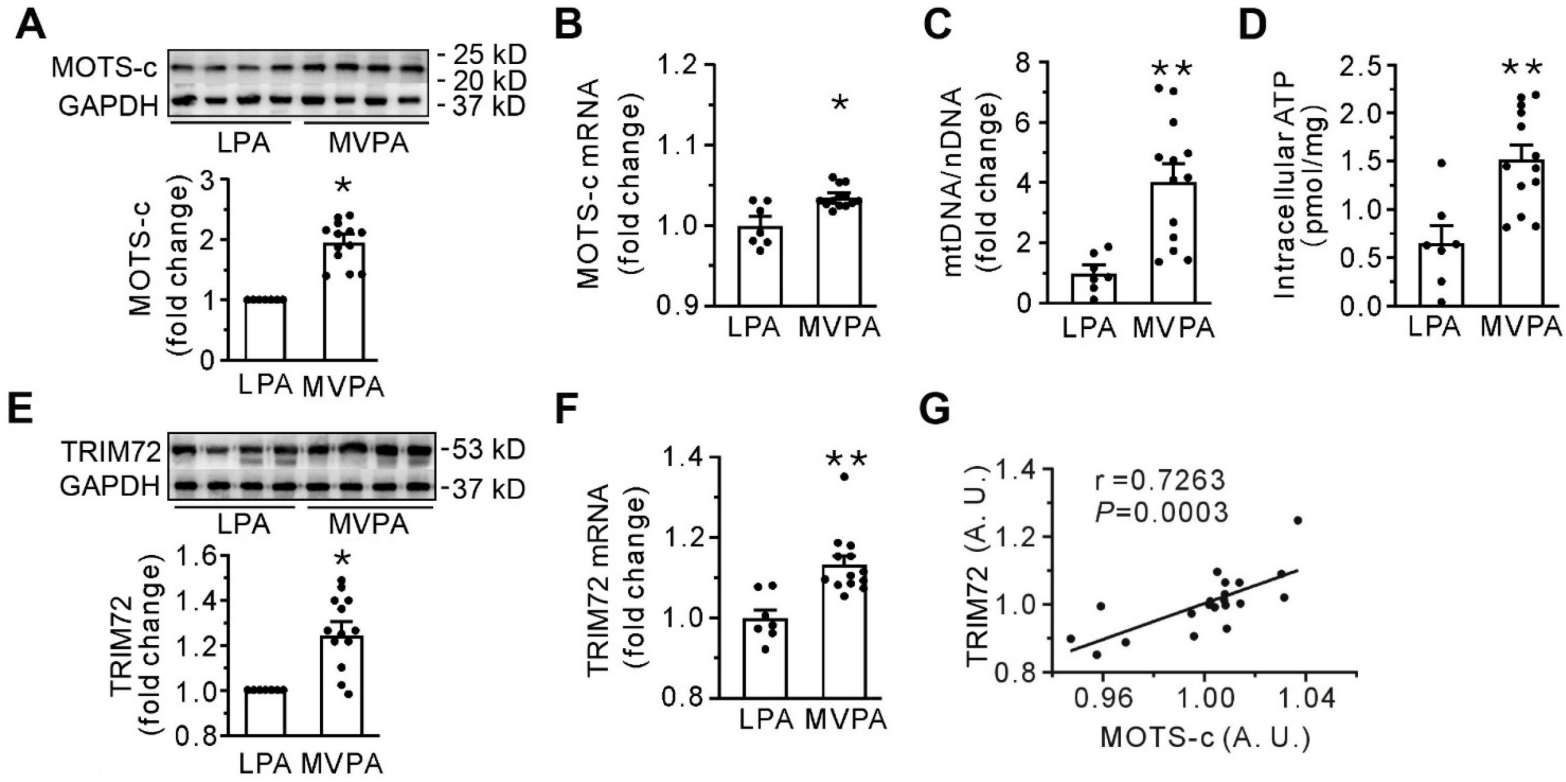
** Moderate to vigorous physical activity (MVPA) increases both MOTS-c and TRIM72 in the skeletal muscle of the human participants. (A)** Representative Western blotting along with densitometric analysis of MOTS-c. **(B)** qPCR detection of MOTS-c. The results are expressed relative to the light-intensity physical activity (LPA) group. (**C-D.** Grouped results of qPCR for mtDNA and intracellular ATP content. **(E)** Representative Western blotting along with densitometric analysis of TRIM72.** (F)** qPCR analysis of TRIM72. The results are expressed relative to the LPA group. **(G)** A correlation analysis between the abundance of MOTS-c and TRIM72 mRNA. Data were analyzed by the 2^-ΔΔCt^ method, and an arbitrary unit (A. U.) was expressed by a ratio of the value in each sample to the mean value for all the participants. The data are expressed as the mean ± SEM using bars with scatter dot plots. Each dot represents an individual human participant. *P < 0.05, **P < 0.01 *vs.* LPA.

Table 1 presents the demographic parameters, clinical conditions, diseases at admission, profiles of the sample, and physical activity levels in all human participants. Apart from age, there were no differences in these parameters between the moderate to vigorous and the low physical activity groups. Compared with low physical activity (LPA group), the moderate to vigorous physical activity (MVPA group) had a stimulated expression of MOTS-c, abundance of mtDNA, and intracellular ATP content in skeletal muscle (**Figures 1A-D**), indicating the dependence of the level of MOTS-c on mtDNA. Furthermore, moderate to vigorous physical activity also increased the expression of TRIM72 (**Figures 1E-F**), and correlation studies revealed a significant positive relationship between the levels of MOTS-c and TRIM72 (**Figure 1G**). These results indicate that MOTS-c may be involved in membrane repair in human subjects.

### Both moderate-intensity exercise and MOTS-c promotes plasma membrane repair

**Figure 2:**
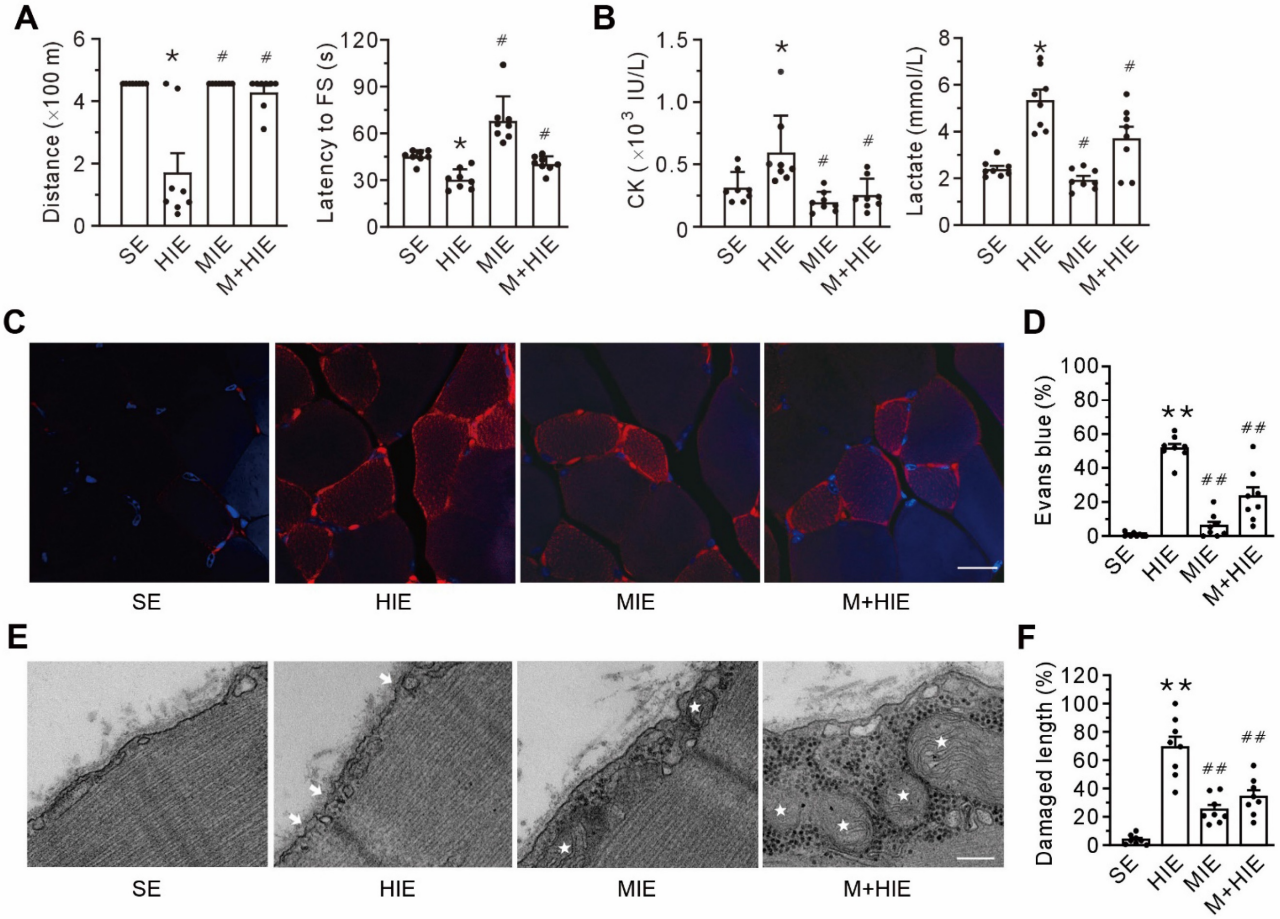
**MOTS-c treatment ameliorates high-intensity exercise-induced membrane disruption. (A)** Treadmill performance. The left panel is the total distance traveled; the right panel is the latency to the first shock (FS) to stimulate running. **(B)** Grouped results of the levels of creatine kinase (CK, left panel) and lactate (right panel) in the plasma. **(C-D)** Representative confocal images of Evans blue staining and group results of the percentage of Evans blue-positive cells. The columns left to right are the sedentary (SE), high-intensity exercise (HIE), moderate-intensity exercise (MIE), and HIE with MOTS-c (HIE+M) groups. Scale bar, 20 μm. **(E-F)** Representative transmission electron micrographs and group results of the length of the damaged membrane. The columns left to right are SE, HIE, MIE, and HIE+M groups. Arrows indicate the nanopores of the plasma membrane; stars symbolize mitochondria. Scale bar, 200 nm. All mice received a 23 m/min exercise challenge at a declination of 15º for 30 min on the day after finishing the exercise regimen. MOTS-c was administered intraperitoneally at a dose of 15 mg/kg/d during acclimation and high-intensity exercise. The data are expressed as the mean ± SEM using bars with scatter dot plots. Each dot represents an individual animal. *P < 0.05, **P < 0.01 *vs.* SE, ^#^*P* < 0.05, ^##^*P* < 0.01 *vs.* HIE. For morphological examination, three separate regions per muscle were examined, and experiments were repeated in 8 animals.

Compared with the sedentary (SE) group, the high-intensity exercise challenge (HIE) group experienced a decline in physical capacity, as evidenced by the decrease in running distance and shortening of the latency to the first shock to stimulate running (**Figure 2A**), as well as an increase in creatine kinase and lactate in the plasma (**Figure 2B**). Furthermore, high-intensity exercise challenge also led to plasma membrane damage, as indicated by the accumulation of intracellular Evans blue (**Figures 2C-D**). TEM corroborated the plasma membrane damage in the HIE group, manifested by the discontinuity of the plasma membrane and the low electron density (**Figures 2E-F**). Of particular interest, both the moderate-intensity exercise (MIE) and MOTS-c treatment (M+HIE) groups blunted all the alterations mentioned above (**Figure 2**). These results pinpoint that plasma membrane integrity is an important prerequisite of physical capacity, which involves MOTS-c.

### MOTS-c serves as a messenger from mitochondria to the plasma membrane

**Figure 3:**
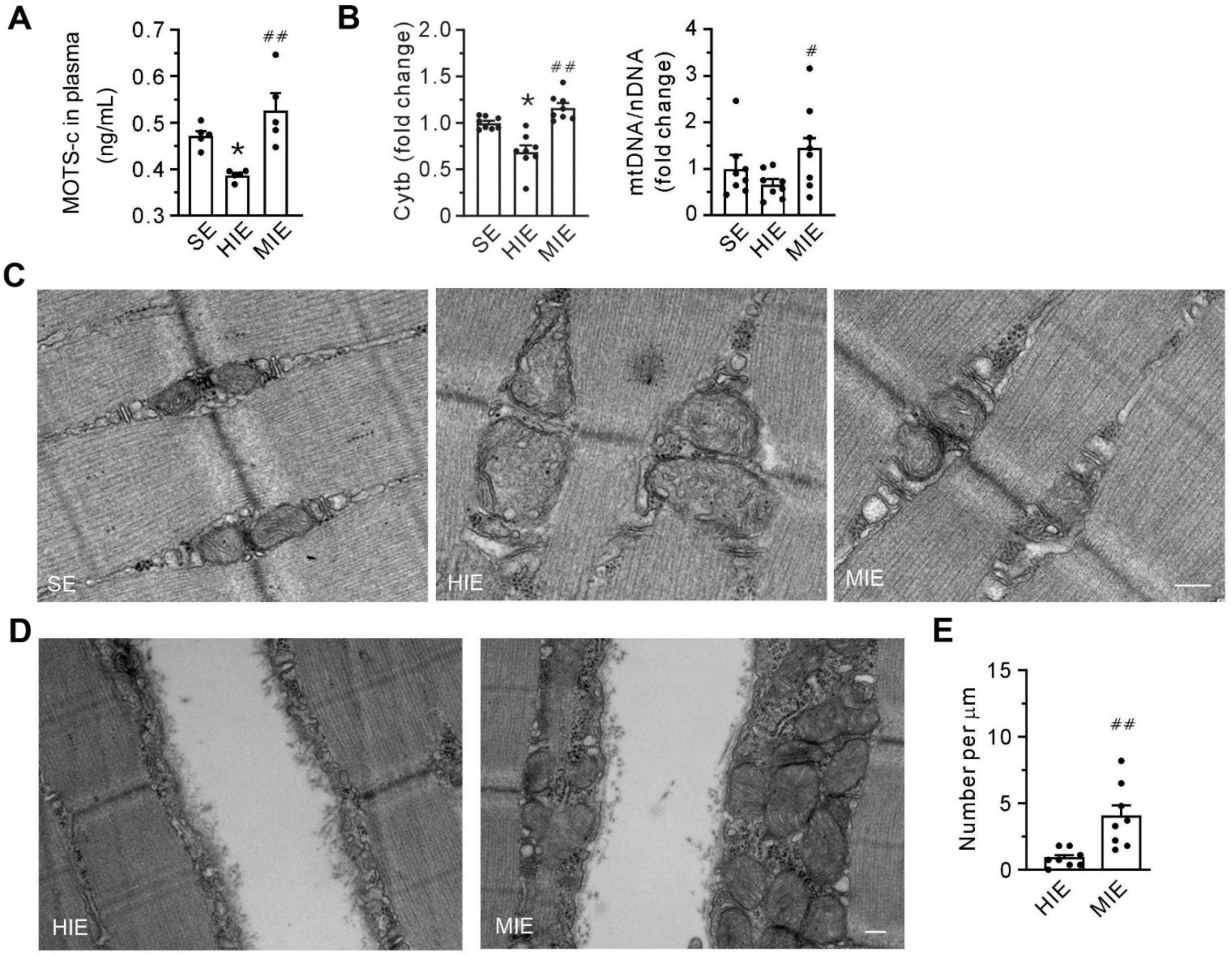
** Moderate-intensity exercise increases the abundance of mitochondria in skeletal muscle and the level of MOTS-c in plasma. (A)** The concentration of MOTS-c in plasma. **(B)** Grouped results of the abundance of mtDNA (Cytb) and the ratio of mtDNA to nuclear DNA (cyclophilin A) with qPCR.** (C)** Transmission electron micrography showing the morphology of the interfibrillar mitochondria. Scale bar, 200 nm. **(D)** Transmission electron micrography showing the mitochondria underneath the plasma membrane. Scale bar, 200 nm. **(E)** Quantification of the number of mitochondria underneath the damaged membrane. The specimen was from gastrocnemius. Three separate regions per muscle were examined, and experiments were repeated in 8 animals. The data are expressed as the mean ± SEM using bars with scatter dot plots. Each dot represents an individual animal. *P < 0.05 *vs.* SE, ^#^*P* < 0.05, ^##^*P* < 0.01 *vs.* HIE.

Compared with SE conditions, HIE was associated with a reduction in plasma levels of MOTS-c (**Figure 3A**). In addition, qPCR data showed that HIE decreased the abundance of cytochrome b mRNA and the ratio of mtDNA/nDNA (**Figure 3B**), indicating a loss of mitochondria. However, all the parameters were increased within the MIE group. Cells from the HIE group exhibited pronounced structural damage to mitochondria, manifested as swelling, reduced density, and loss of matrix and cristae. However, these morphological alterations were almost negligible in cells from the MIE group (**Figure 3C**). Notably, in contrast to the HIE group, MIE induced the accumulation of abundant mitochondria underneath the injured membrane (**Figures 3D-E**). These results suggest that mitochondria are involved in plasma membrane repair, with MOTS-c mediating this effect.

### MOTS-c promotes plasma membrane deformability, which involves TRIM72

**Figure 4:**
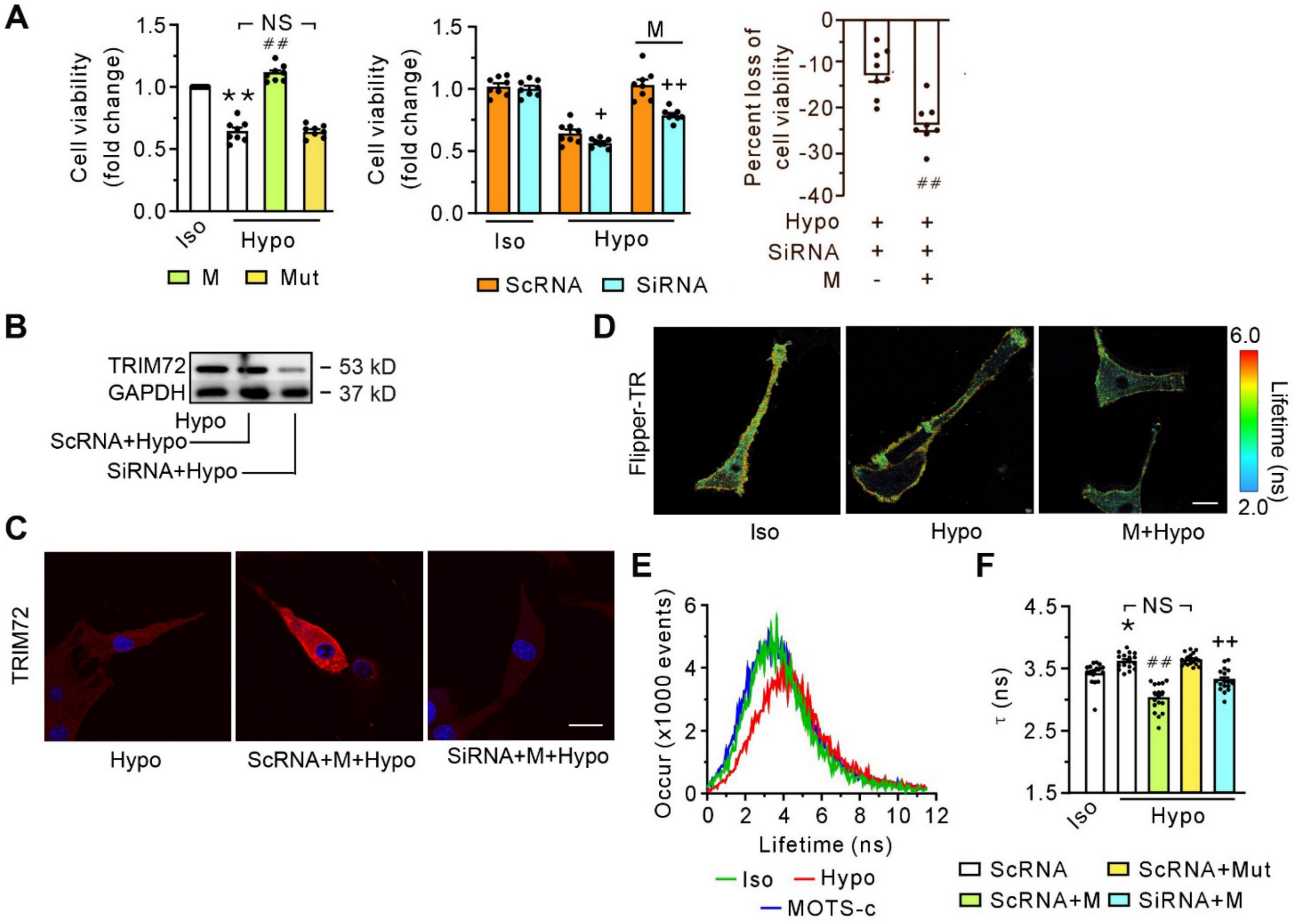
** SiRNA-TRIM72 blocks the beneficial effects of MOTS-c in cell viability and membrane deformability. (A)** Grouped results of the cell viability without (left panel) or with (middle and right panels) siRNA-TRIM72. The blocking potency of siRNA-TRIM72 is presented in the right panel, in which the loss of cell viability is calculated as a percentage of the difference between siRNA- and scRNA-treated group relative to the value in the corresponding scRNA-treated group, as shown in the middle panel. The cells were exposed to isotonic or hypotonic solution for 30 min. **(B)** Representative immunoblot of TRIM72. **(C)** Representative confocal images with or without siRNA-TRIM72 treatment. The differentiated C2C12 cells were transfected with siRNA-TRIM72 for 6 h, and scramble RNA (scRNA) was used as a control. Scale bar 20 μm. **(D)** Fluorescence lifetime τ1 images of Flipper-TR under isotonic solution or exposed to hypotonic solution in the presence or absence of 1 μM MOTS-c. The color bar corresponds to the lifetime in nanoseconds. MOTS-c at 1 μM was administered 5 min before and together with hypotonic solution exposure. Scale bar, 100 μm.** (E)** Representative time course tracing of lifetime τ1 when C2C12 cells were exposed to hypotonic solution in the absence or presence of 1 μM MOTS-c. **(F)** Grouped results of lifetime τ1 mean values. The data are expressed as the mean ± SEM using bars with scatter dot plots. Each dot represents one round of experiment or an individual cell. *P < 0.05, **P < 0.01 vs. Iso, ^##^*P* < 0.01 vs. corresponding group without MOTS-c. ^++^*P* < 0.01 vs. the corresponding group with scRNA. NS, no significant difference. Iso, Isotonic solution; Hypo, Hypotonic solution; M, MOTS-c; Mut, mutant of MOTS-c; SiRNA, siRNA-TRIM72.

Exposure of differentiated C2C12 cells to hypotonic solution for 30 minutes caused a decrease in cell viability (**Figure 4A**, left panel). MOTS-c, but not its mutant, alleviated cell injury caused by the hypotonic solution (**Figure 4A**, left panel). The effectiveness of MOTS-c in preventing hypotonic solution-induced injury was significantly reduced by the application of siRNA (**Figure 4A**, middle panel), which diminished the content of TRIM72 (**Figure 4B**). Of particular interest, the reduction in protective efficacy of MOTS-c, due to siRNA-targeting TRIM72, was more pronounced in the MOTS-c treated cells compared to cells not treated with MOTS-c (**Figure 4A**, right panel). siRNA-TRIM72 alone slightly but significantly increased cell susceptibility to hypotonic injury (**Figure 4A**, middle panel). Immunostaining revealed that MOTS-c enhanced the trafficking of TRIM72 to the plasma membrane, which was blocked by siRNA-TRIM72 (**Figure 4C**). In addition, upon exposure to the hypotonic solution, the cells exhibited a prolongation of the fluorescence lifetime τ1 of Flipper-TR, an indicator of membrane tension. MOTS-c, but not its mutant, antagonized this harmful effect caused by the hypotonic solution (**Figures 4D-F**). Of note, this effect of MOTS-c was also blocked by siRNA-TRIM72 (**Figure 4F**). These results indicate that MOTS-c improves membrane integrity or deformability via facilitating the translocation of TRIM72 or increasing TRIM72 expression.

### AMPK is not a prerequisite component in MOTS-c-triggered membrane repair

**Figure 5:**
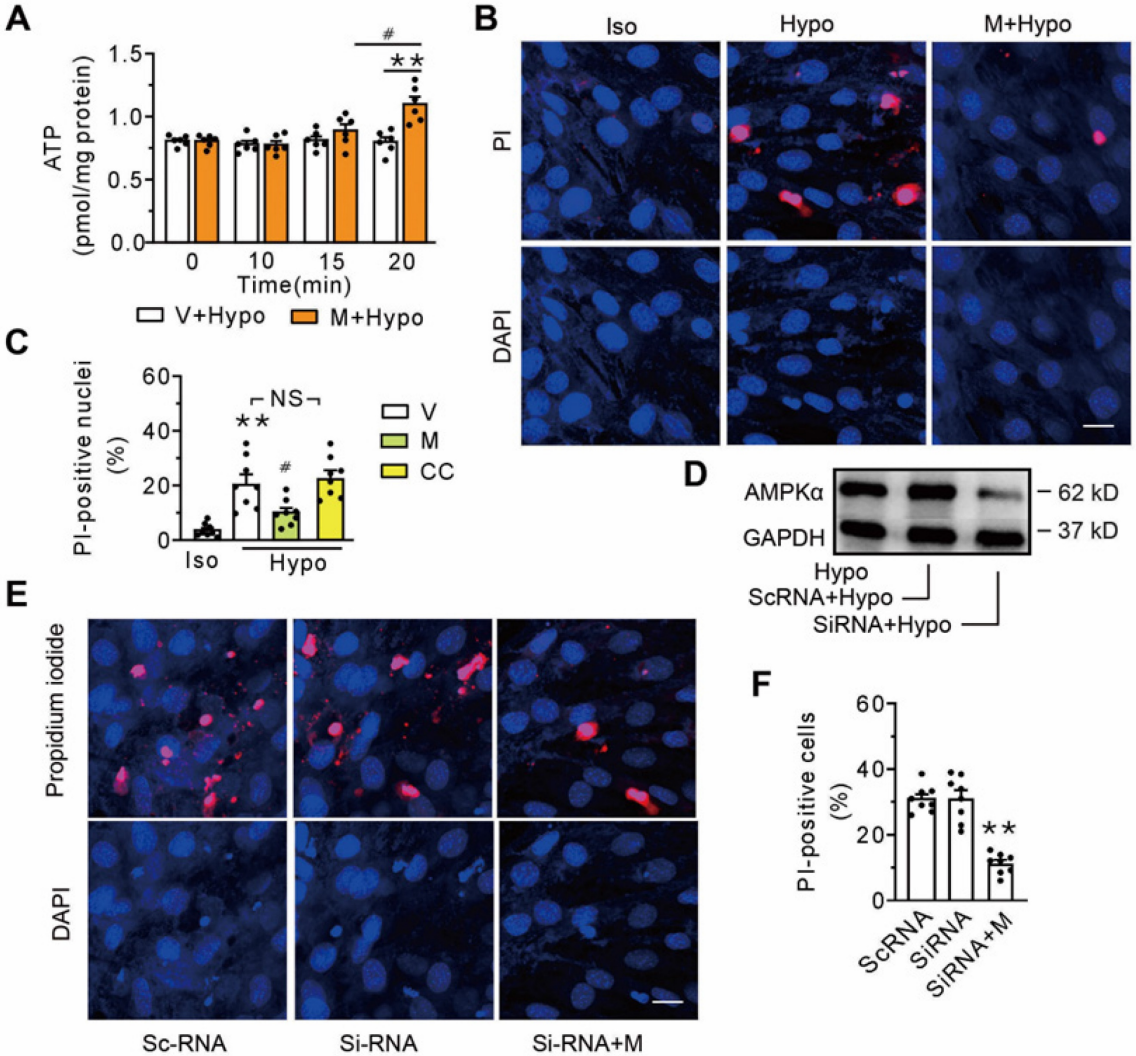
** Inhibition of AMPK fails to antagonize MOTS-c-triggered membrane repair. (A)** Measurement of intracellular ATP at different times after addition of MOTS-c. The cells were exposed to hypotonic solution in the absence or presence of 1 μM MOTS-c. ^#^*P* < 0.05, **P < 0.01. **(B-C)** Representative confocal images and group results on the percentage of propidium iodide-positive nuclei after treatment with MOTS-c or 10 μM Compound C. The cells were exposed to hypotonic solution for 15 min in the presence of 100 μg/ml propidium iodide. MOTS-c and Compound C were administered 5 min before hypotonic solution exposure. **P < 0.01 *vs.* Iso, ^#^*P* < 0.05 *vs.* Hypo+V. NS, no significant difference.** (D)** Representative immunoblot of AMPKα. **(E-F)** Representative confocal images and group results on the percentage of propidium iodide-positive nuclei after treatment with siRNA-AMPKα. **P < 0.01 *vs.* siRNA. Scramble RNA (scRNA) was used as a control. Scale bar 20 μm. The data are expressed as the mean ± SEM using bars with scatter dot plots. Each dot represents one round of experiment. V, vehicle (saline); Hypo, Hypotonic solution; PI, propidium iodide; M, MOTS-c; CC, Compound C.

AMPK, an energy sensor known to stimulate ATP generation, has been documented to be a downstream target of MOTS-c. To determine whether AMPK was involved in membrane repair triggered by MOTS-c, a time course study of the cells exposed to the hypotonic solution was performed. Compared with the vehicle-treated group, intracellular ATP did not increase until 20 min following the addition of 1 μM MOTS-c (**Figure 5A**). Thus, we determined the percentage of the propidium iodide-positive nuclei at 15 min after administering MOTS-c. **Figures 5B and C** show that MOTS-c reduced the percentage of positive nuclei, an effect not mirrored by the addition of Compound C, an inhibitor of AMPK. Furthermore, treatment with siRNA-AMPK failed to extinguish MOTS-c-triggered membrane repair (**Figures 5D-F**). Collectively, these results indicate that AMPK is not a prerequisite component in MOTS-c-triggered membrane repair.

### The improvement of the plasma membrane integrity depends on the trafficking of the MOTS-c/TRIM72 complex

**Figure 6:**
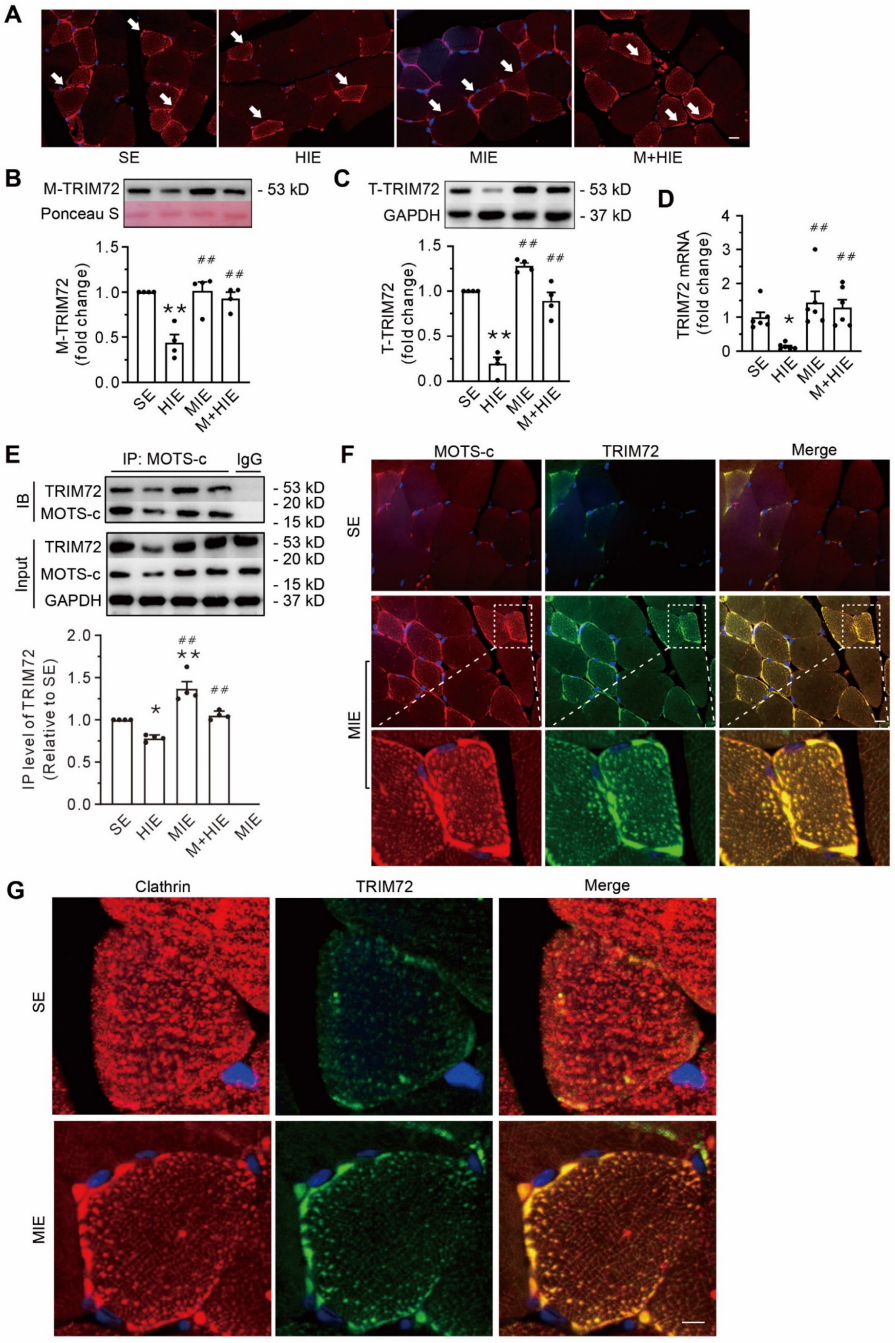
** Replenishment of MOTS-c increases the trafficking of TRIM72 to the plasma membrane via interacting with TRIM72 and enhancing the expression of TRIM72. (A)** Representative immunofluorescence staining of TRIM72 in each group. Arrows indicate the membrane-positive staining. Scale bar, 20 μm. **(B-C)** Representative Western blotting of TRIM72 in the membrane fraction (M-TRIM72) and in total lysates (T-TRIM72) along with densitometric analysis. The data are expressed as the fold change relative to the sedentary group. **(D)** Grouped results of qPCR measurements of TRIM72 mRNA. **(E)** Representative Co-IP and input blots from tissue lysates, along with grouped analysis of total binding amounts of TRIM72 targeted to MOTS-c, which are indicated by IP level of TRIM72, and expressed with respect to that in sedentary group (SE). IB, immunoblot; IP, immunoprecipitation.** (F)** Immunofluorescence staining showing the colocalization of MOTS-c (red) and TRIM72 (green) in the plasma membrane and cytosol. Scale bar, 20 μm. The bottom exhibited a 3.5-fold enlargement from the dashed areas in the moderate-intensity exercise group. **(G)** Immunofluorescence staining showing both clathrin and TRIM72-positive particles. Scale bar, 5 μm. The data are expressed as the mean ± SEM using bars with scatter dot plots. Each dot represents an individual anmial. *P < 0.05, **P < 0.01 *vs.* SE, ^##^*P* < 0.01 *vs.* HIE. For immunofluorescence analysis, three separate regions per muscle were examined, and experiments were repeated in 6 animals.

Relative to sedentary conditions, HIE decreased the TRIM72 protein in both the plasma membrane fraction and the total cell lysate fraction. HIE also decreased the abundance of TRIM72 mRNA, which MIE did not (**Figure 6A-D**). Furthermore, MOTS-c treatment blunted these alterations caused by HIE (**Figure 6A-D**).

Co-IP data showed that compared with SE, HIE decreased the total binding amounts of TRIM72 targeted to MOTS-c, whereas MIE increased them (**Figure 6E**). Moreover, MOTS-c treatment antagonized the diminished effects of HIE (**Figure 6E**). Of note, the binding capability of TRIM72 to MOTS-c, which was expressed with IP level of TRIM72 relative to the MOTS-c IP blot, revealed no differences among groups (**Suppl. Data 1**). Confocal images revealed the colocalization of MOTS-c and TRIM72 not only in the plasma membrane but also in the cytosol, which was particularly obvious in cells from the MIE group (**Figure 6F**). In addition, muscle cells in the SE group displayed an even distribution of the vesicle marker clathrin and a nearly undetectable amount of TRIM72 in the cytosol (**Figure 6G**). In contrast, MIE elicited the expression of TRIM72, which was colocalized with clathrin and was more apparent in the plasma (**Figure 6G**). These results indicate that an association of MOTS-c with TRIM72, followed by the fusion of intracellular TRIM72-coated vesicles to the plasma membrane, is an important step in MOTS-c triggered-membrane repair.

**Figure 7:**
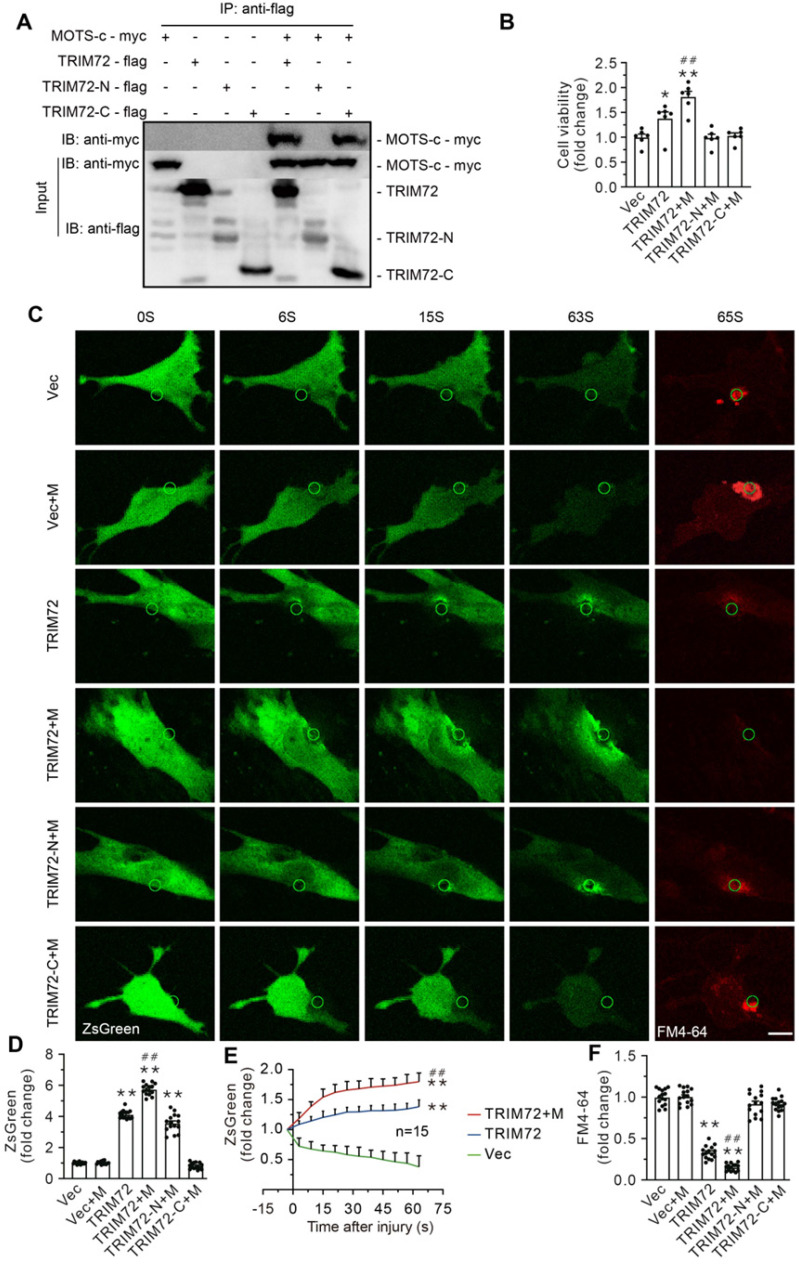
**MOTS-c interacted with TRIM72 at C-terminus. (A)** Representative Co-IP of TRIM72-flag and MOTS-c-myc in lysates of HEK293T cells overexpressing MOTS-c and TRIM72, N-terminus (TRIM72-N) or C-terminus (TRIM72-C). **(B)** Grouped results on cell viability. Before exposing to hypotonic solution, cells were transfected with vector (Vec), MOTS-c (M), TRIM72, TRIM72-N and TRIM72-C. **(C)** Representative time-lapse images showing membrane repair process. C2C12 cells were transfected with ZsGreen-tagged TRIM72, and damaged using a pulsed laser in the presence of the FM4-64 fluorescent dye (red color). The green circle lines indicate the location of the damaged membrane. The injury intensity is indicated by FM4-64 accumulation. Scale bar, 10 μm. **(D)** Quantification of the ZsGreen-TRIM72 signal at the injury site. **(E)** Plot showing membrane repair kinetics monitored by ZsGreen intensity. **(F)** Quantification of the FM4-64 accumulation at the injury site at the end of experiments. The data are expressed as the mean ± SEM using bars with scatter dot plots. Each dot represents one round of experiment or an individual cell. *P < 0.05, **P < 0.01 *vs.* Vec. ^##^*P* < 0.01 *vs.* TRIM72.

TRIM72 comprises a tripartite motif domain (TRIM) at its N-terminus and a PRY domain followed by a SPRY domain at its C-terminus. Protein-protein docking analysis using ClusPro server4-8 (https://cluspro.org) revealed that the SPRY domain at C-terminus serves as a potential binding site linking TRIM72 to MOTS-c. To determine the TRIM72-binding site of MOTS-c, HEK293T cells were co-transfected with the myc-tagged pLV4Itr-mCherry-CMV vector, which overexpressed MOTS-c, and the flag-tagged pLV4Itr-ZsGreen-CMV vector, which overexpressed either the full-length TRIM72 or its N-terminus or C-terminus segments. Cell lysates were immunoprecipitated with anti-flag antibody, and immunoblots exhibited flag-positive bands, indicating that immunoprecipitation pulldown worked (**Suppl. Data 2**). Furthermore, myc-tagged MOTS-c was detected in cells carrying full-length TRIM72 and its C-terminus, illustrating that MOTS-c interacts with TRIM72 C-terminus but not N-terminus (**Figure 7A**). Overexpressing full-length TRIM72 preserved cell viability against hypotonic injury (**Figure 7B**). Of particular interest, cells that overexpressed both MOTS-c and full-length TRIM72 were more resistant to the hypotonic challenge than cells that solely overexpressed the full-length TRIM72 (**Figure 7B**). However, this phenomenon disappeared in cells transfected with either the N-terminus or C-terminus alone (**Figure 7B**). These results suggest that an association of MOTS-c with TRIM72 at the C-terminus activates TRIM72 to repair injured membrane.

To evaluate the role of MOTS-c in the dynamic process of membrane repair in living cells, the plasma membrane of C2C12 cells was mechanically damaged using a two-photon laser in the presence of the FM4-64 fluorescent dye, an indicator of membrane injury (**Figure 7C-F & Suppl. Data 4**). Cells carrying with full-length TRIM72 exhibited profound membrane repair, indicated by higher green fluorescent intensity and less FM4-64 fluorescent dye at the injured site (**Figure 7C-F**). Of particular interest, MOTS-c boosted membrane repair, manifested as enhanced trafficking of TRIM72 and negligible FM4-64 at the injured area (**Figure 7C-F**). In contrast, MOTS-c failed to facilitate membrane repair in cells carrying with either TRIM72 N-terminus or TRIM C-terminus, although TRIM72 N-terminus itself had an ability to translocate to injured membrane (**Figure 7C-F & Suppl. Data 3**).

### The interaction between MOTS-c and lipids contributes to the translocation of TRIM72 to the membrane

**Figure 8:**
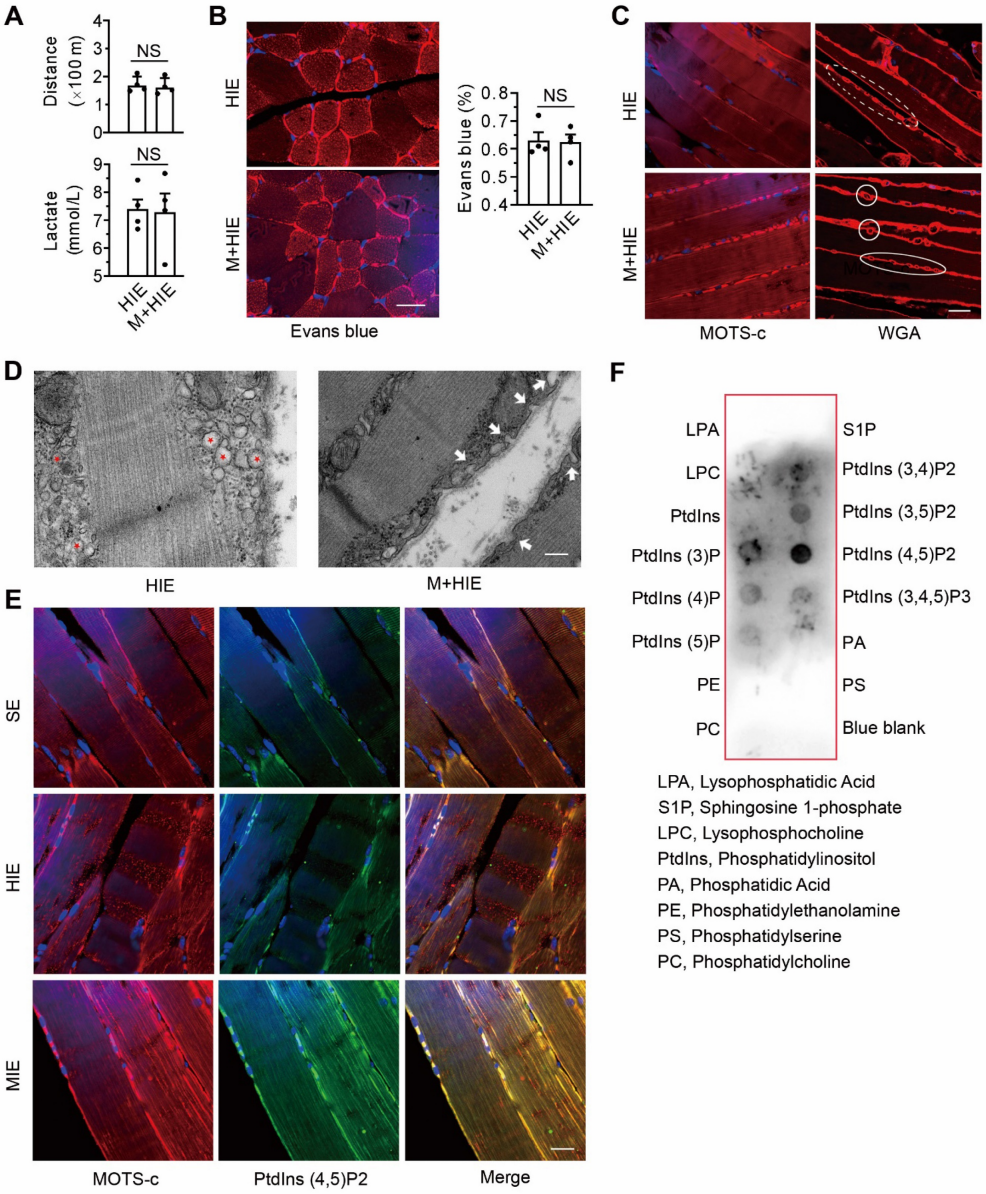
**Replenishment of MOTS-c fails to improve membrane integrity in TRIM72^-/-^ mice but stimulates the translocation of intracellular vesicles to the membrane via binding to lipids. (A)** Group results of total distance traveled and lactate in plasma at the end of exercise challenge in TRIM72^-/-^ mice. **(B)** Representative images of fluorescent probing of Evans blue in TRIM72^-/-^ mice. Scale bar, 20 μm. **(C)** Representative immunostaining of MOTS-c and WGA in TRIM72^-/-^ mice. Scale bar, 20 μm. Solid circles indicate WGA-positive vesicles passing across membrane, which is different from that in the dashed circle. **(D)** Representative transmission electron micrographs in TRIM72^-/-^ mice. Red stars denote the aggregation of many intracellular vesicles beneath the plasma membrane in the HIE group. White arrows indicate the fusion of vesicles to the plasma membrane in the M+HIE group. The gastrocnemius muscles were exercised for examination. Scale bar, 200 nm. **(E)** Representative immunofluorescent double staining of MOTS-c (red) and PtdIns (4,5) P2 (green). Scale bar, 20 μm. **(F)** Dot blotting showing the interactions between MOTS-c and membrane lipids. All TRIM72 KO mice received high-intensity exercise for 7 days. The exercise protocols, the regimen for treadmill performance examination, and MOTS-c treatment were identical to those in Figure 2. The data are expressed as the mean ± SEM using bars with scatter dot plots. Each dot represents an individual animal. NS, no significant difference. For immunofluorescence analysis, three separate regions per muscle were examined, and experiments were repeated in 6 animals.

As expected, replenishing MOTS-c in TRIM72^-/-^ mice failed to improve their physical capacity, as evidenced by running distance and the level of lactate in plasma (**Figure 8A**). It also failed to rescue plasma membrane injury, as indicated by the fluorescence intensity of intracellular Evans blue (**Figure 8B**). These results confirm that the improvement of membrane integrity by MOTS-c is dependent on TRIM72.

Compared with the saline-treated TRIM72^-/-^ mice, the muscles of MOTS-c-treated mice exhibited stronger MOTS-c-positive staining on the plasma membrane (**Figure 8C**). Of particular interest, after MOTS-c treatment, more WGA-positive vesicle-like structures passed across the plasma membrane (**Figure 8C**), indicating the fusion of the vesicles to the plasma membrane upon MOTS-c stimulation. These phenomena were confirmed by TEM. As shown in Figure 8D, the knockout of TRIM72 resulted in the aggregation of many vesicles under the plasma membrane, whereas MOTS-c treatment facilitated the fusion of the vesicles to the membrane (**Figure 8D**). Confocal images showed stronger staining of double MOTS-c and PtdIns (4,5) P2 within the plasma membrane of cells from the MIE group than from the HIE group (**Figure 8E**). As expected, dot blot analysis revealed an interaction of MOTS-c with PtdIns (4,5) P2 (**Figure 8F**). Thus, the interaction between MOTS-c and lipids may be another important step contributing to the MOTS-c-triggered membrane repair.

### Treatment with MOTS-c increases TRIM72 trafficking and attenuates myocardial ischemia/reperfusion injury

**Figure 9:**
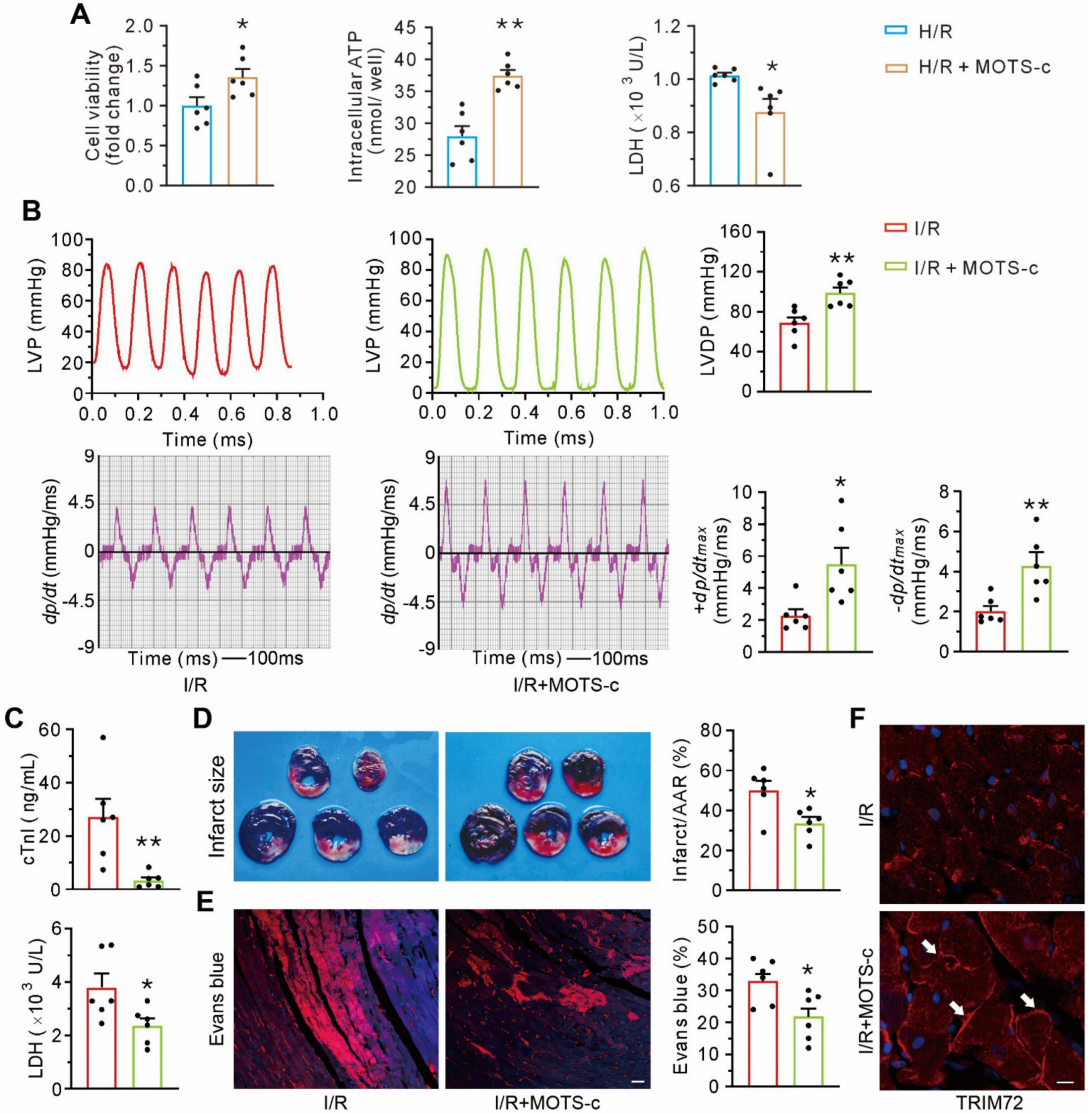
** MOTS-c blunts myocardial ischemia/reperfusion injury. (A)** Grouped results on effects of MOTS-c on cell viability, ATP content, and LDH leak in hypoxia-reoxygenated neonatal rat cardiomyocytes. **(B)** Representative tracings of left ventricular pressure (LVP) and the corresponding ± dp/dtmax, and grouped results of left ventricular developed pressure (LVDP) and ± dp/dtmax. **(C)** Grouped results of cardiac troponin I (cTnI) and lactate dehydrogenase (LDH) in plasma at the end of reperfusion.** (D)** Representative images of heart slices stained with triphenyl tetrazolium chloride (TTC) and grouped results of infarct size, which was expressed as a percentage of area at risk (AAR). **(E)** Representative confocal images showing the intracellular accumulation of Evans blue and grouped results of the positive area which was expressed as a percentage of the total area. Scale bar, 25 μm. **(F)** Representative images of fluorescent probing of TRIM72. White arrows indicate an increase of membrane- recruited TRIM72 in the I/R+MOTS-c group. Scale bar, 20 μm. The data are expressed as the mean ± SEM using bars with scatter dot plots. Each dot represents one round of experiment or an individual animal. *P < 0.05, **P < 0.01 *vs.* H/R or I/R. H/R, hypoxia/reoxygenation. I/R, ischemia/reperfusion.

Compared with the control group, MOTS-c protected cultured neonatal ventricular myocytes against hypoxia/reoxygenation-induced injury, manifested as an increase in cell viability and ATP content and a decrease in LDH leakage (**Figure 9A**). *In viv*o studies revealed that MOTS-c treatment preserved myocardial systolic and diastolic function, as reflected by an improvement of LVDP, ±dp/dtmax (**Figure 9B**). Furthermore, MOTS-c decreased the level of cardiac troponin I and lactate dehydrogenase in plasma after myocardial ischemia/reperfusion (**Figure 9C**). The hearts from MOTS-c-treated animals showed a reduction in infarct size (**Figure 9D**) and less accumulation of intracellular Evans blue, indicated by the red fluorescence signal (**Figure 9E**), but an increase in the translocation of TRIM72 to the plasma membrane (**Figure 9F**). These results indicate that MOTS-c/TRIM72 trafficking is an effective approach for alleviating ischemia/reperfusion injury.

## Discussion

The most interesting observations of this study are as follows: (1) MIE was shown to increase mitochondrial abundance and promote the production of MOTS-c, thereby enhancing cellular membrane integrity; (2) MOTS-c improved plasma membrane integrity by interacting with TRIM72 and facilitating the trafficking of TRIM72, which was similar to the treatment with MIE; (3) inhibition of AMPK failed to block MOTS-c-triggered membrane repair; (4) MOTS-c targeted PtdIns (4,5) P_2_ in the plasma; (5) the level of MOTS-c was positively correlated with TRIM72 in human; (6) the intervention of MOTS-c/TRIM72 pathway was an effective approach against myocardial ischemia/reperfusion injury. Our study for the first uncovered MOTS-c as a messenger in mitochondria-triggered membrane repair. The actions of MOTS-c include: (1) improving membrane integrity, which depends on the trafficking of TRIM72; (2) enhancing the fusion of vesicles to membrane, which depends on plasma lipids, rather than TRIM72. More importantly, our results provide the evidence that stimulating the pathway of MOTS-c/TRIM72 not only rescues high-intensity exercise-induced skeletal muscle injury but also remedies the function of organs by promoting membrane repair in clinical settings, such as myocardial ischemia/reperfusion injury.

Emerging evidence reveals that mitochondria serve as a cellular hub for sensing environmental changes, including mechanical and chemical stimuli. Accordingly, mitochondria release some signals to regulate key cellular processes that have a significant impact on health span and lifespan. A previous study demonstrated that reactive oxygen species served as messengers from mitochondria to locally trigger guanosine triphosphatase (GTPase) RhoA activation, actin polymerization, and plasma membrane repair 52. In this study, (1) MIE promoted the accumulation of mitochondria beneath the damaged membrane, (2) MIE increased the production of MOTS-c, and (3) replenishment of MOTS-c, the mitochondria-derived peptide, enhanced the trafficking of TRIM72 to the plasma membrane and improved membrane integrity. These data identify MOTS-c as a new signal from mitochondria to regulate plasma membrane repair.

In the present study, MOTS-c antagonized high-intensity exercise-induced skeletal muscle damage, evidenced by an improvement in treadmill running performance and a decrease in the content of creatine kinase and lactate in plasma membrane damage. In this scenario, MOTS-c improved membrane integrity, reflected by a reduction in intracellular accumulation of Evans blue and the continuity of plasma membrane under electron transmission micrograph examination. Similarly, MOTS-c alleviated the membrane damage caused by hypotonic solution, manifested by a decrease in intracellular accumulation of propidium iodide. More importantly, MOTS-c blunted the increase of the membrane tension elicited by hypotonic solution, as reflected by the immunofluorescence indicator Flipper-TR. These results are consistent with previous findings that membrane damage is a primary cause of cellular injury 1-3, 6. Of note, our data for the first provide evidence that the beneficial effects of MOTS-c on membrane integrity are associated with an improvement in membrane deformability.

MOTS-c is involved in multiple pathophysiological processes, i.e., improving insulin sensitivity by modulating glucose and fatty acid metabolism, transmitting signals from the mitochondria to the nucleus, ameliorating skeletal muscle atrophy, and increasing physical activity 28, 30, 33. Furthermore, AMPK, a key molecule responsible for cell metabolism, is believed an essential downstream messenger to mediate these effects 28. In this study, treatment with MOTS-c did not drive ATP generation in cells exposed to hypotonic solution at 15 min. What is more, inhibition of AMPK with Compound C and siRNA failed to block MOTS-c-triggered membrane repair, reflected by the percentage of propidium iodide-positive cells. Taking into consideration the finding that TRIM72 was a target of MOTS-c in the process of membrane repair, we conclude that MOTS-c has a novel role in participating in membrane repair, and AMPK is not a prerequisite ingredient in this scenario. In addition to the pentose phosphate pathway (involving activation of AMPK), RNA-sequencing and metabolomics analysis data revealed re-programming/adaption of amino acid metabolism, oxidase stress response, nuclear transport, and longevity-related pathway activation following MOTS-c treatment, which involves heat shock factor 1 33. Further research is warranted to investigate whether these processes are involved in membrane repair.

Here, we observed that MOTS-c and TRIM72 co-localized in both the plasma membrane and the cytosol. Moreover, MIE increased the plasma membrane content of the particles containing both TRIM72 and MOTS-c, as well as the particles containing both TRIM72 and clathrin, a marker of intracellular vesicles. Additionally, the depletion of TRIM72 and interference of siRNA-TRIM72 diminished the membrane integrity improvement induced by MOTS-c, as evidenced by intracellular accumulation of Evans blue and propidium iodide. These data indicate that TRIM72 is a critical effector for MOTS-c-triggered maintenance of membrane integrity. TRIM72 possesses a TRIM domain at its N-terminus and a SPRY domain at its C-terminus, both of which are involved in interactions with other proteins 53. In the present study, Co-IP data showed an interaction of MOTS-c with TRIM72 C-terminus, but not N-terminus. Of particular interest, dynamic membrane repair assay showed that MOTS-c enhanced the trafficking of the full-length TRIM72 to injured membrane. Furthermore, co-overexpression of MOTS-c and full-length TRIM72 was more resistant to hypotonic challenge. In contrast, these phenomena were lost in cells overexpressing either C-terminus, or N-terminus. Based on these data, we postulate that the association of MOTS-c with TRIM72 at C-terminus activates the TRIM72 to repair damaged membrane. In addition, it has to be noted that Co-IP data revealed an interaction between MOTS-c and TRIM72 C-terminus. However, MOTS-c failed to facilitate the trafficking of TRIM72 C-terminus to injured membrane. We interpret these findings to indicate that the function of MOTS-c depends on an intact form of TRIM72.

An interesting observation to note here is that the total binding amounts of TRIM72 targeted to MOTS-c increase following MIE or HIE with MOTS-c (Figure 6E), although the binding capability does not alter (**Suppl. Data 1**). Thus, it is likely that the abundance of MOTS-c may be an important factor to facilitate the translocation of TRIM72 to membrane. Furthermore, taking into consideration the observations on the dynamic membrane repair process, we suggest that MOTS-c accelerates the transportation of the TRIM72-contained vesicles. In addition, our data demonstrated that treatment with MOTS-c increases the expression of TRIM72. These results corroborate the viewpoint that MOTS-c transmits the signal from the mitochondria to the nucleus to activate the stress-responsive transcriptional response 30 and, more importantly, suggest that an increase in TRIM72 content is an important prerequisite conferring the enhanced trafficking of TRIM72 by MOTS-c. Apart from TRIM72, other important effectors involved in plasma membrane repair include dysferlin, annexins, caveolin 3, and endosomal sorting complexes required for transport proteins 7, 25, 49, and further investigation is warranted to explore the relationship between MOTS-c and these proteins. It is well-known that MOTS-c is a short peptide. The predicted molecular weight of MOTS-c is about 2 kD. Here, Western blot data revealed a molecular weight between 10-20 kD in the mouse, which was consistent with previous findings 27, 33, and a molecular weight between 20-25 kD in human skeletal muscle. One of explanations for these differences may be due to the posttranscriptional modifications, such as glycosylation 54, which deserve further studies.

In this study, immunostaining showed co-localization of MOTS-c and PtdIns(4,5)P_2_. Furthermore, dot blot data revealed that MOTS-c interacted with PtdIns(4,5)P_2_. These data are consistent with previous finding that PtdIns(4,5)P_2_ is involved in intracellular trafficking and membrane dynamics^55^, ^56^, and raise the possibility that PtdIns(4,5)P_2_ is an important ingredient in MOTS-c-elicited membrane repair. Our data are in contrast to a previous report that TRIM72 binds to phosphatidylserine 21. Thus, further studies are warranted to investigate the impacts of MOTS-c/PtdIns(4,5)P_2_ interaction on the cellular process of phosphatidylserine-TRIM72 complex-mediated cell membrane repair. Here, compared with saline-treated TRIM72^-/-^ animals, the muscle from MOTS-c-treated ones exhibited an increase in the fusion of intracellular vesicles to the plasma membrane (Figure 8), along with a decrease in intracellular vesicles (Figure 8). These data suggest that the motor proteins responsible for vesicle translocation/anchoring, i.e., Rab GTPase, kinesin, and dynein, may be alternative targets of MOTS-c to facilitate the vesicle trafficking 57. Further studies are deserved to gain insight into the role of motors in this scenario. We also propose that two steps are involved in MOTS-c-triggered membrane repair: the first step relies on TRIM72-mediated membrane integrity repair, and the second step relies on vesicle translocation/anchoring.

In this study, we observed that MOTS-c had a protective effect on heart, as evidenced by an increase of cell viability, a reduction in infarct size and improvement in cardiac function. These results are consistent with previous findings that MOTS-c enhanced heart performance during exercise training 58. As documented previously, membrane injury is a salient cause during myocardial ischemia/reperfusion injury and other diseases 6, 7, 10, 11. Furthermore, MOTS-c increased the translocation of TRIM72 to the plasma membrane, and blocked membrane disruption. These results suggest that MOTS-c-mediated promotion of membrane repair could be a potential therapeutic approach for preserving the function of organs. Here, one point needs to mention is that besides implicating in membrane repair, TRIM72 acts as an E3 ubiquitin ligase, which is related to insulin resistance 59, 60. In contrast, MOTS-c promotes metabolic homeostasis via up-regulating AMPK 28. Thus, we suggest that application of MOTS-c may exert dual actions by accelerating TRIM72-mediated membrane repair and inhibiting the activity of E3 ubiquitin ligase caused by TRIM72 simultaneously.

Previous studies demonstrated that a higher level of overexpressed TRIM72 was a prominent determinant in protecting the heart, kidney, lungs, and skeletal against noxious stimulus 14, 19, 20, 22. In this study, we only detected the level of TRIM72 under basal conditions. Our data showed a mild increase in TRIM72 protein and mRNA in the moderate to vigorous physical activity group of human participants. These results suggest that moderate to vigorous physical activity may endow the subjects a more potential to stimulate TRIM72 expression under stressful conditions to improve membrane integrity. Here, we only observed that the level of MOTS-c was positively correlated with TRIM72 in human participants. Due to unavailable data on membrane integrity, caution should be taken to conclude that the MOTS-c/TRIM72 pathway is linked to cell membrane repair in human beings. More clear evidence is needed to unravel the clinical relevance of the MOTS-c/TRIM72 pathway in membrane damage-related diseases.

In conclusion, this study has identified a novel pathway involving MOTS-c/TRIM72 in mediating mitochondria-triggered plasma membrane repair, which involves MOTS-c/TRIM72-coated vesicles trafficking, and the interaction of MOTS-c with TRIM72 and plasma lipids. Moreover, our findings provide a new strategy to rescue organ function by facilitating MOTS-c-mediated membrane repair in clinical settings. A profound understanding of the function of MOTS-c and its underlying mechanisms will help to pave the way for its clinical application.

## Supplementary Material

Supplementary figures and data.

## Figures and Tables

**Table 1:** Baselines of the included participants

Characteristics		LPA (n=7)	MVPA (n=13)	*P* value
Demographic profile				
	Age	65.3±2.7	50.3±3.5	< 0.05
	Sex (man, %)	4 (57.1)	7 (53.8)	> 0.05
	Weight (kg)	70.4±4.8	70.8±3.1	> 0.05
	BMI (kg/m^2^)	24.8±0.8	26.1±1.1	> 0.05
Clinical profile				
	Hypertension (n, %)	3 (42.9)	5 (38.5)	> 0.05
	Coronary heart disease (n, %)	0	0	
	Osteoporosis (n, %)	6 (85.7)	5 (38.5)	> 0.05
	Depression (n, %)	0	0	
Disease at admission				
	Osteoarthritis (n, %)	3 (42.9)	3 (23.1)	> 0.05
	Femoral head necrosis (n, %)	3 (42.9)	5 (38.5)	> 0.05
	Sport injury (n, %)	1 (14.2)	5 (38.5)	> 0.05
Specimen profile				
	Gluteus maximus (n, %)	4 (57.1)	7 (53.8)	> 0.05
	Quadriceps femoris (n, %)	3 (42.9)	3 (23.1)	> 0.05
	Sartorius muscle (n, %)	0	3 (23.1)	> 0.05
