# Supplementary material for: Mitochondria-encoded peptide MOTS-c participates in plasma membrane repair by facilitating the translocation of TRIM72 to membrane
Source: Theranostics. 2024 Aug 19;14(13):5001–21. doi: 10.7150/thno.100321 (PMC11388074; doi:10.7150/thno.100321)
Supplement: Supplementary file 1 — Supplementary figures and data. [file thnov14p5001s1.zip › supplementary/Suppl. Data 1-3.docx]

**SUPPLEMENTARY INFORMATION**

**Mitochondria-encoded peptide MOTS-c participates in plasma membrane repair by facilitating the translocation of TRIM72 to membrane**

Hong JIA^1,4, †^, Lyu-Chen ZHOU^1,^ ^†^, Yong-Feng CHEN^1, †^, Wei ZHANG^3^, Wei QI^1^, Peng WANG^1^, Xiao HUANG^1^, Jian-Wei GUO^1^, Wai-Fang HOU^1^, Ran-Ran ZHANG^1^, Jing-Jun ZHOU^2 *^, Da-Wei ZHANG^1 *^

^1^Department of Orthopedics, Xijing Hospital, Fourth Military Medical University, Xi’an 710032, China; ^2^Department of Physiology, Southwest Medical University, Luzhou 646000, China; ^3^Department of Neurology, Tangdu Hospital, Fourth Military Medical University, Xi’an 710032, China; ^4^Western Theater Command Center for Disease Control and Prevention, Lanzhou 730020, China

^†^These authors contributed to this work equally.

^*^Correspondence: [dw_zhang7721@163.com](mailto:dw_zhang7721@163.com), or to [jing-jun.zhou@swmu.edu.cn](mailto:jing-jun.zhou@swmu.edu.cn).

**
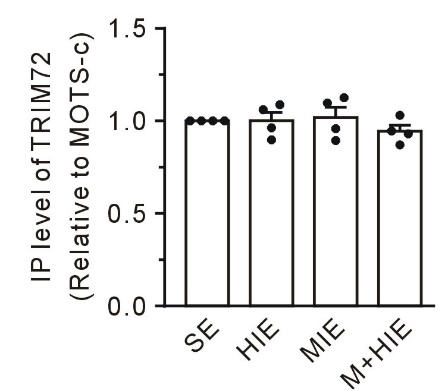
**

**Suppl. Data 1.** Group results of the binding capability of TRIM72 targeted to MOTS-c. The densitometric ratio of TRIM72 to MOTS-c in immunoblots after immunoprecipitation (Figure 6E) is calculated, and then normalized to SE. The value is indicated as the binding capability. The data are expressed as the mean ± SEM using bars with scatter dot plots. Each dot represents an individual animal. There are no differences among groups.

**
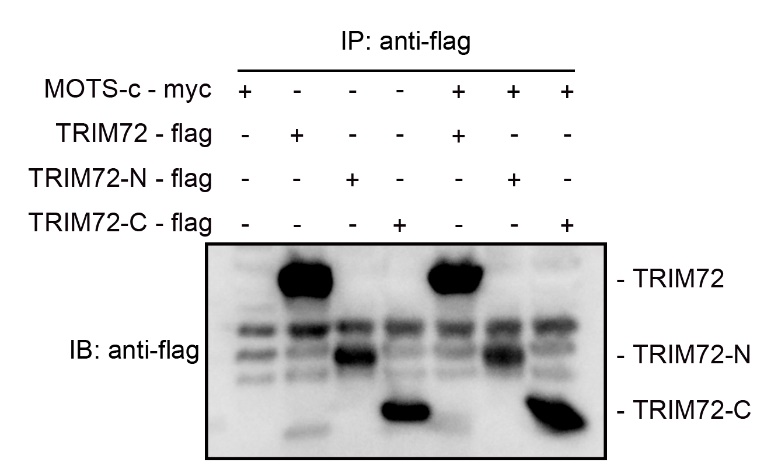
**

**Suppl. Data 2.** Representative flag immunoblots after immunoprecipitation. HEK293T cells were co-transfected with the myc-tagged pLV4Itr-mCherry-CMV vector, which overexpressed MOTS-c, and the flag-tagged pLV4Itr-ZsGreen-CMV vector, which overexpressed either the full-length TRIM72 or its N-terminus or C-terminus segments. The cell lysates were immunoprecipitated with anti-flag antibodies.


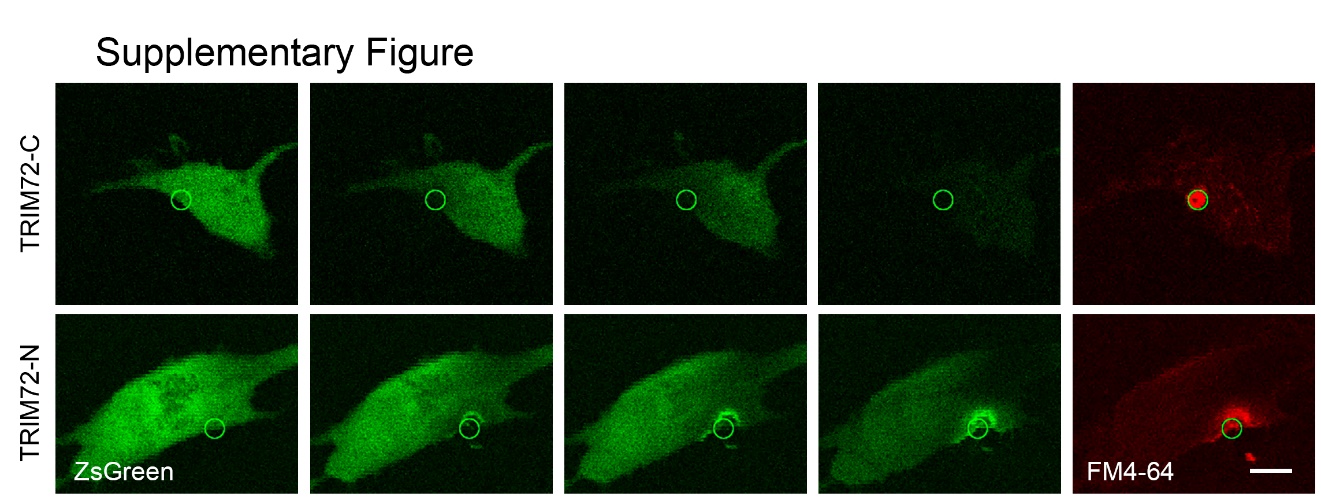


**Suppl. Data 3.** Representative time-lapse images showing membrane repair process. C2C12 cells were transfected with ZsGreen-tagged TRIM72 C terminus (TRIM72-C) or N terminus segments (TRIM72-N), and damaged using a pulsed laser in the presence of the FM4-64 fluorescent dye (red color). The green circle lines indicate the location of the damaged membrane. The injury intensity is indicated by FM4-64 accumulation. Scale bar, 10 μm.
