# Supplementary material for: Mitochondria-encoded peptide MOTS-c participates in plasma membrane repair by facilitating the translocation of TRIM72 to membrane
Source: Theranostics. 2024 Aug 19;14(13):5001–21. doi: 10.7150/thno.100321 (PMC11388074; doi:10.7150/thno.100321)
Supplement: Supplementary file 1 — Supplementary figures and data. [file thnov14p5001s1.zip › supplementary/Suppl.Date 4/Description of Suppl. Data 4.docx]

**Description of Suppl. Data 4**

**Description:** Representative videos (×.wmv) and images (×.jpg) showing the dynamic membrane repair process (ZsGreen) and membrane damage (accumulation of red fluorescent dye FM4-64). The file names were compiled as follows.

**Note:** G and R respectively represent ZsGreen (green color) and FM4-64 (red color); +M means treatment with MOTS-c.

**1 Vec-G.wmv** and **1 Vec-R.jpg**

refer to a cell carrying vector without MOTS-c treatment.

**2 Vec+M-G.wmv** and **2 Vec+M-R.jpg**

refer to a cell carrying vector with MOTS-c treatment.

**3 TRIM72-G.wmv** and **3 TRIM72-R.jpg**

refer to a cell carrying TRIM72 without MOTS-c treatment.

**4 TRIM72+M-G.wmv** and **4 TRIM72+M-R.jpg**

refer to a cell carrying TRIM72 with MOTS-c treatment.

**5 TRIM72-C-G.wmv** and **5 TRIM72-C-R.jpg**

refer to a cell carrying TRIM72 C terminus without MOTS-c treatment.

**6 TRIM72-C+M-G.wmv** and **6 TRIM72-C+M-R.jpg**

refer to a cell carrying TRIM72 C terminus with MOTS-c treatment.

**7 TRIM72-N-G.wmv** and **5 TRIM72-N-R.jpg**

refer to a cell carrying TRIM72 N terminus without MOTS-c treatment.

**8 TRIM72-N+M-G.wmv** and **6 TRIM72-N+M-R.jpg**

refer to a cell carrying TRIM72 N terminus with MOTS-c treatment.
