# Supplementary figures and images for: Mitochondria-encoded peptide MOTS-c participates in plasma membrane repair by facilitating the translocation of TRIM72 to membrane
Source: Theranostics. 2024 Aug 19;14(13):5001–21. doi: 10.7150/thno.100321 (PMC11388074; doi:10.7150/thno.100321)

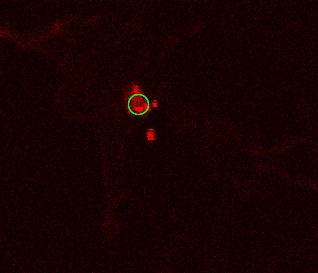

Supplement: Supplementary file 1 — Supplementary figures and data. [file thnov14p5001s1.zip › supplementary/Suppl.Date 4/1-2 Vec-R.jpg]

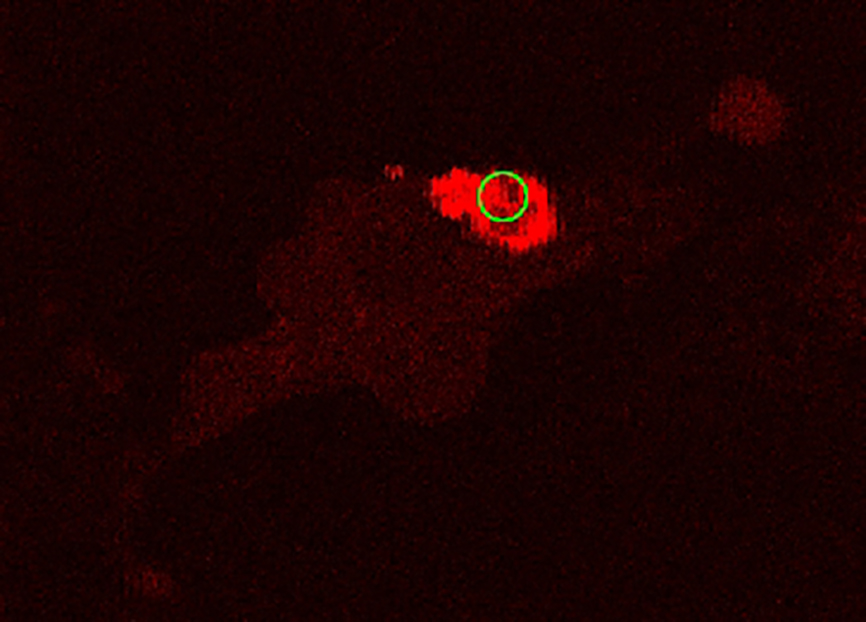

Supplement: Supplementary file 1 — Supplementary figures and data. [file thnov14p5001s1.zip › supplementary/Suppl.Date 4/2-2 Vec+M-R.jpg]

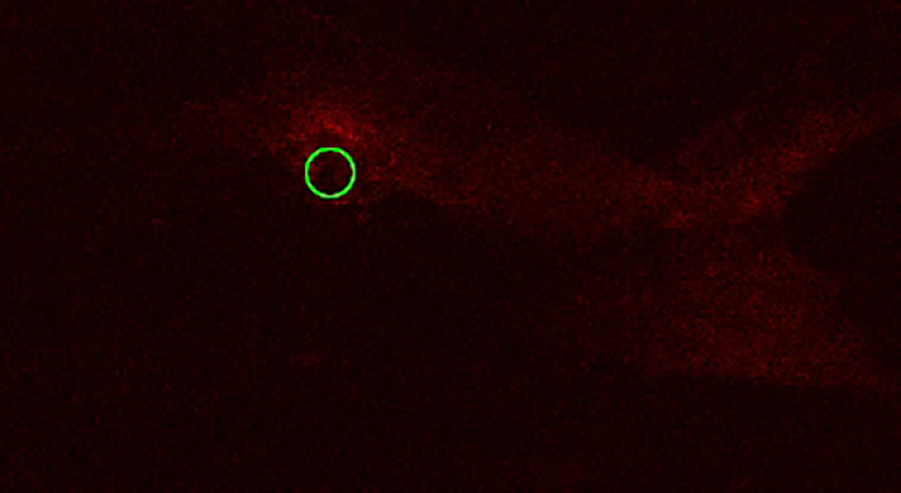

Supplement: Supplementary file 1 — Supplementary figures and data. [file thnov14p5001s1.zip › supplementary/Suppl.Date 4/3-2 TRIM72-R.jpg]

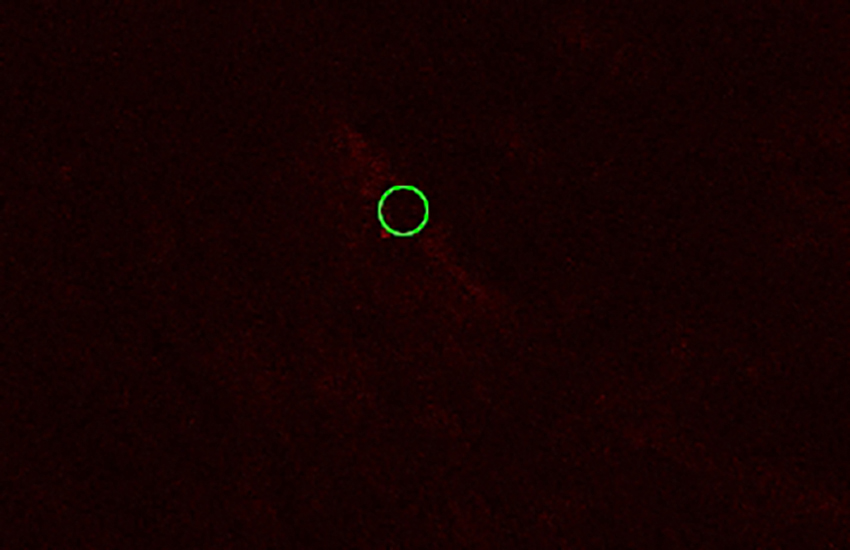

Supplement: Supplementary file 1 — Supplementary figures and data. [file thnov14p5001s1.zip › supplementary/Suppl.Date 4/4-2 TRIM72+M-R.jpg]

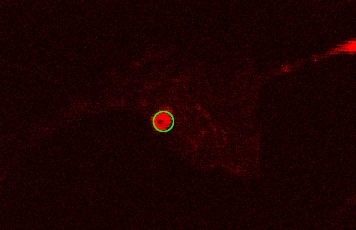

Supplement: Supplementary file 1 — Supplementary figures and data. [file thnov14p5001s1.zip › supplementary/Suppl.Date 4/5-2 TRIM72-C-R.jpg]

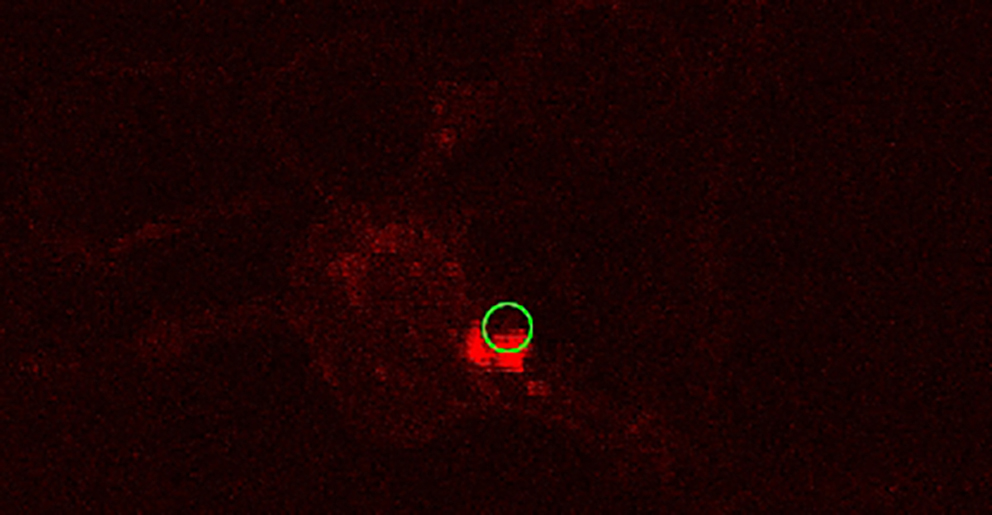

Supplement: Supplementary file 1 — Supplementary figures and data. [file thnov14p5001s1.zip › supplementary/Suppl.Date 4/6-2 TRIM72-C+M-R.jpg]

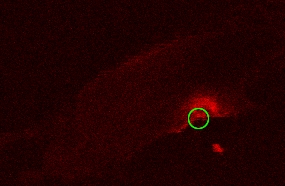

Supplement: Supplementary file 1 — Supplementary figures and data. [file thnov14p5001s1.zip › supplementary/Suppl.Date 4/7-2 TRIM72-N-R.jpg]

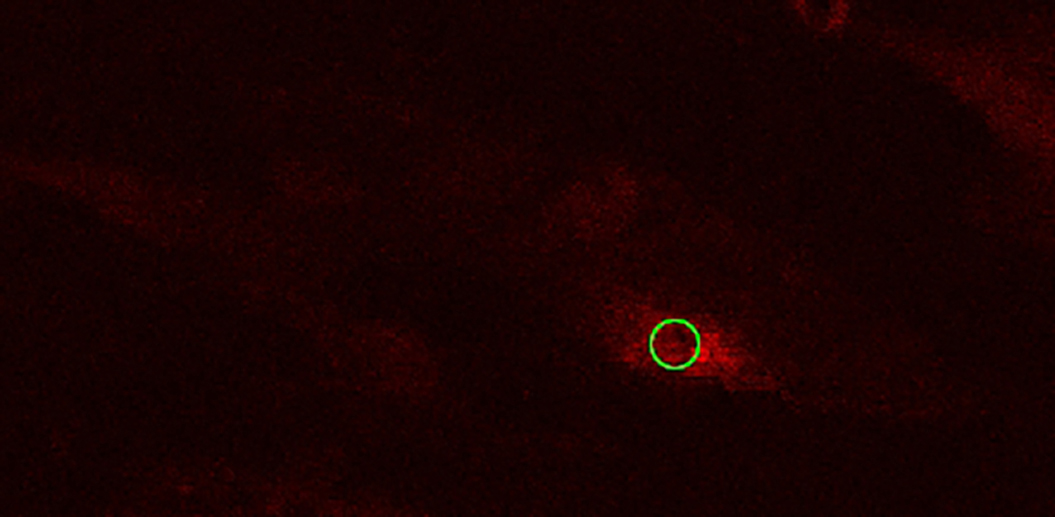

Supplement: Supplementary file 1 — Supplementary figures and data. [file thnov14p5001s1.zip › supplementary/Suppl.Date 4/8-2 TRIM72-N+M-R.jpg]
